# Supplementary material for: Dyslipidemia-associated natural IgM improves oncolytic virus TILT-123 efficacy through antibody-dependent enhancement in solid tumors
Source: Mol Ther. 2026 Jan 20;34(5):3056–76. doi: 10.1016/j.ymthe.2026.01.019 (PMC13154276; doi:10.1016/j.ymthe.2026.01.019)
Supplement: Supplementary file 1 [file mmc1.pdf]

YMTHE, Volume *34*

Supplemental Information

Dyslipidemia-associated natural IgM improves oncolytic virus TILT-123 efficacy through antibody-dependent enhancement in solid tumors

James Hugo Armstrong Clubb, Santeri Artturi Pakola, Sakari Joenväärä, Tatiana Viktorovna Kudling, Tiialotta Tohmola, Victor Arias, Elise Jirovec, Mirte van der Heijden, Dafne Carolina Alves Quixabeira, Annukka Pasanen, Lyna Haybout, Nea Ojala, Saru Basnet, Arianna Eleuteri, Julia Davila Ferrero, Stella Hirvenoja, Inge Marie Svane, Johanna Mäenpää, Katriina Jalkanen, Matthew Stephen Block, Tuomo Alanko, Tine Monberg, Sanae Zahraoui, Susanna Grönberg-Vähä-Koskela, Natasha Salmelin, Claudia Kistler, Riikka Havunen, Suvi Sorsa, João Manuel dos Santos, Victor Cervera-Carrascon, Anna Kanerva, Otto Hemminki, Risto Renkonen, and Akseli Hemminki

Table S1. Data file containing source data used in this study. See separate excel file.

Table S2. Patient characteristics and responses. F = female, M = male. NSCLC = non-small cell lung cancer, HSGOC = High Grade Serous Ovarian Cancer, NA = not available, PD = progressive disease, SD = stable disease, PR = partial response, PMD = progressive metabolic disease, SMD = stable metabolic disease, PMR = partial metabolic response, * = patient alive at data-cutoff.

Patient ID	Cohort	Sex	ECOG	Tumour Type	Stage	No. prior systemic treatments	RECIST 1.1	PET Response	Overall Survival (Days)
20202	1	F	1	NSCLC	IV	5	PD	PMR	102
20203	1	F	1	Melanoma of unknown primary	IV	7	NA	NA	66
20204	2	M	0	Myxoid liposarcoma	IV	5	PD	SMD	821
20205	2	F	1	Ovarian Serous Cystadenocarcinoma	IV	14	NA	NA	70
20101	2	F	1	Breast carcinoma	IV	7	NA	NA	56
20206	2	F	1	HGSOC	IIIC	16	PD	SMD	192
20103	3	M	0	Anaplastic Thryoid carcinoma	IV	1	PR	PMR	814*
20102	3	M	1	Rhabdomyosarcoma	IV	3	NA	NA	118
20211	3	F	1	Cutaneous melanoma	IV	3	PD	PMD	377
20212	4	F	1	Leiomyosarcoma	IV	4	PD	PMD	557
20104	4	M	1	Leiomyosarcoma	IV	6	PD	SMD	206
20107	4	M	0	Chondrosarcoma	IV	2	NA	NA	104
20213	5	M	1	Neuroendocrine carcinoma of the bladder	IV	3	NA	NA	94
20108	5	F	1	Adenoid cystic carcinoma	IV	0	SD	SMD	221
20214	5	F	1	Mucinous carcinoma of the appendix	IV	6	NA	NA	109
20216	6	F	1	Cutaneous melanoma	IV	2	NA	NA	104
20109	6	F	0	Leiomyosarcoma	IV	5	NA	NA	109
20217	6	F	1	Myxoid liposarcoma	IV	4	PD	PMD	131
20219	6	M	1	High grade mucoepidermoid carcinoma of the parotid gland	IV	2	PD	PMD	239
20111	6	F	0	High grade serous carcinoma of the peritoneum	IV	15	NA	NA	196

Table S3. PET criteria.

Complete metabolic response (CMR)	Complete resolution of FDG activity within measurable lesions and all reliably assessable lesions to background levels. No new FDGavid lesions in pattern typical of cancer*. Lymph nodes may remain metabolically active due to immune response (activated lymphocytes take up FDG).
Partial metabolic response (PMR)	> 30 % reduction in FDG activity measured as the summed SUVmax of measurable lesions (up to five lesions, max 2/organ). No new FDGavid lesions in pattern typical of cancer*.
Minor metabolic response (MMR)	10-29 % reduction in FDG summed SUVmax. No new FDG-avid lesions in pattern typical of cancer*.
Stable metabolic disease (SMD)	0-9 % reduction or up to < 30 % increase in FDG summed SUVmax. No new FDG-avid lesions in pattern typical of cancer*.
Progressive metabolic disease (PMD)	≥ 30 % increase in FDG summed SUVmax in pattern typical of tumor, or new clearly FDG-avid clinically significant lesions in pattern typical of cancer*. Increase in metabolic activity in lymph nodes should not result in PMD if no progression is detected elsewhere, since it might reflect immunological activation and not progression.
*"clinically significant lesions in pattern typical of cancer" are defined as lesions associated with a CT abnormality most consistent with cancer (≥ 2 cm in diameter), and clearly not because of inflammation of infection or related to treatment response.	

Table S4. IGLV8-61 containing clones detected from full length BCR sequencing. Including patient ID, day of sample collection, heavy chain variable (VH) gene, joining heavy (JH) gene, diversity heavy chain (DH), heavy chain CDR3 sequence, variable light (VL) gene, joining light (JL) gene, light chain CDR3 sequence and Chain (isotype). NA indicates where information was not available. IGLV8-61*01 is highlighted in the green column. BL = baseline.

#	Patient ID	Day	VH gene	DH gene	JH gene	Heavy chain CDR3	VL gene	JL gene	Light chain CDR3	Constant region
1	20103	64	IGHV3-23*01	IGHD2-8*02	IGHJ6*02	ARDGGVSYYGMDV	IGLV8-61*01	IGLJ3*02	VLYMGSGIWV	NA
2	20103	64	IGHV4-38-2*02	IGHD6-6*01	IGHJ4*02	ARDPYSSSSAAFHY	IGLV8-61*01	IGLJ3*02	VLYMGSGISV	IGHM
3	20103	64	IGHV3-48*03	IGHD3-22*01	IGHJ4*02	ARAGEATYYYDSSGYYGLHNMYYFDY	IGLV8-61*01	IGLJ3*02	VLYMGSGINWV	IGHM
4	20103	64	IGHV3-30-3*01	IGHD2-15*01	IGHJ6*02	ARDQAPYCSGGSCYDASYYYYGMDV	IGLV8-61*01	IGLJ3*02	VLYMGSGISV	IGHM
5	20103	64	IGHV4-61*01	IGHD2-21*02	IGHJ4*02	ATFATALPGAYFDY	IGLV8-61*01	IGLJ2*01	VLYMNDGVVL	IGHM
6	20211	BL	IGHV3-11*05	IGHD4-17*01	IGHJ4*02	ARLDLFYGDHFDY	IGLV8-61*01	IGLJ3*02	VLYMGSGPHWV	IGHM
7	20211	64	IGHV3-21*01	NA	IGHJ3*02	ARVRDPDAFDI	IGLV8-61*01	IGLJ2*01	LLYLGSGML	IGHD
8	20211	64	IGHV4-61*02	IGHD3-3*01	IGHJ4*02	ARTTIFGVAIDY	IGLV8-61*91	IGLJ3*02	VLYMGSGINWV	IGHG1
9	20212	64	IGHV3-74*01	IGHD2-15*01	IGHJ2*01	ARDSGGWYFDL	IGLV8-61*01	IGLJ3*02	VLYMGSGIWV	IGHM
10	20217	BL	IGHV3-21*01	IGHD6-6*01	IGHJ4*02	ARGSEAARPAEIDY	IGLV8-61*01	IGLJ2*01	LLYLGSGML	IGHG1
11	20217	BL	IGHV3-30*18	IGHD3-22*01	IGHJ3*02	AKDNYYEDAFDI	IGLV8-61*01	IGLJ2*01	LLYLGSGML	NA
12	20217	64	IGHV3-49*04	NA	IGHJ1*01	TRDHWH	IGLV8-61*01	IGLJ2*01	LLYLGSGML	IGHM
13	20219	64	IGHV3-49*04	IGHD4-11*01	IGHJ6*02	TRDPESYSNPWPSADLVSHYYGMDV	IGLV8-61*01	IGLJ2*01	LLYLGSGML	IGHD
14	20219	64	IGHV3-23*01	NA	IGHJ4*02	ANRNYYFVY	IGLV8-61*01	IGLJ1*01	GLYMGSGVSV	IGHM
15	20219	64	IGHV4-30-4*09	IGHD4-11*01	IGHJ4*02	ARVRIYSDFYFDY	IGLV8-61*01	IGLJ3*02	VLYMGSDTWV	IGHM
16	20219	64	IGHV3-33*01	IGHD3-16*01	IGHJ3*02	ARDSLHSNYVYGTQFQHERIFDI	IGLV8-61*01	IGLJ3*02	VLYMGSGIWV	IGHD

Table S5. Flow cytometry panel used in this study including target, clone, fluorochrome, catalogue number and supplier.

Target	Clone	Fluorochrome	Catalogue number	Supplier
CD3	SP34-2	BV786	563918	BD
CD5	UCHT2	APC-Cy7	563516	BD
CD8	SKI	BV510	563919	BD
CD14	M5E2	RB705	757509	BD
CD19	HIBI9	BV650	302238	Biolegend
CD27	M-T271	AF700	356416	Biolegend
CD38	HIT2	BV711	563965	BD
CD43	1G10	BV421	562916	BD
CD204	U23-56	RB613	758361	BD
FCMR	HM7	APC	398104	Biolegend
IgM	G20-127	PE	555783	BD
IgD	1A6-2	RB780	569140	BD

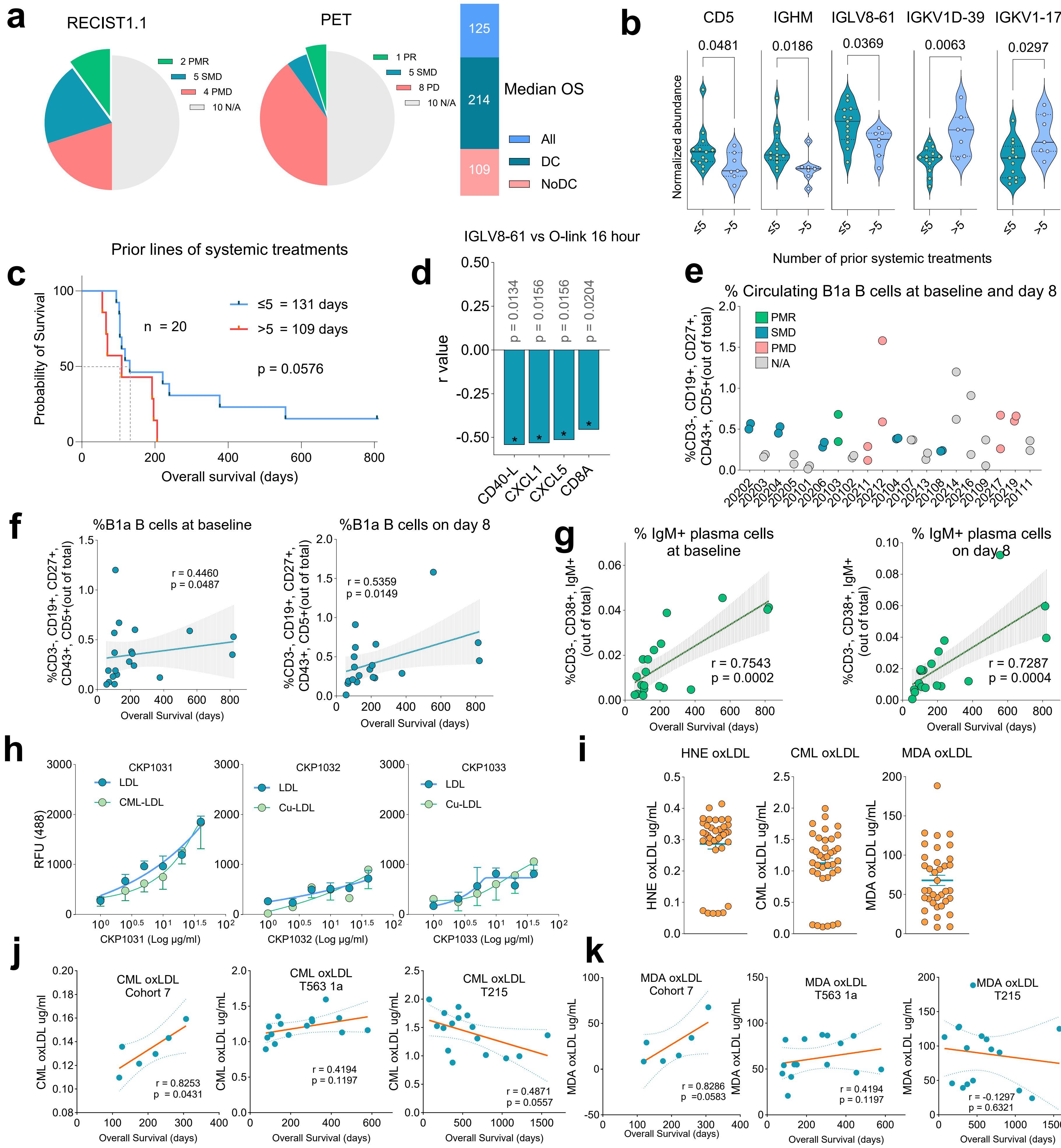

Figure S1. a. Summary of responses in the TUNIMO (T115) monotherapy trial according RECIST1.1, PET criteria and median overall survival for all patients, patients with DC and no DC. b. Group analysis identifying immunoglobulins most significantly associated with patients having more than five versus five or fewer prior lines of systemic treatment. c. Kaplan-Meier curve illustrating OS stratified by patients with more than five versus five or fewer prior lines of systemic treatment (n = 20). Statistical significance was assessed using the log-rank (Mantel-Cox) test. d. Significant correlations between IGLV8-61 abundance and NPX values of immuno-oncology-related proteins on day 8, as measured by Olink analysis. e. Flow cytometry summary showing the percentage of circulating B1a B cells ($CD3^-$, $CD19^+$, $CD27^+$, $CD43^+$, $CD5^+$) among total cells in T115 patients at baseline and day 8, with data points color-coded by best response as defined by PET criteria. f. Correlation between the percentage of circulating B1a B cells at baseline and day 8 with OS. g. Correlation between the percentage of circulating IgM+ plasma cells at baseline and day 8 with OS. h.Direct ELISA performed using IgM clones CPK1031, CPK1032, and CPK1033, with native LDL and CML modified oxLDL. i. Quantification of baseline serum concentrations of HNE oxLDL, CML oxLDL, and MDA oxLDL in patients from cohorts T115 (Cohort 7), T215, and T563 (1a). j-k. Correlation of CML oxLDL and MDA oxLDL levels with OS in patients from the TUNIMO Cohort 7, PROTA (T563 1a), and TUNITIL (T215). Cohort 7 n = 6, T563 1a n = 15, T215 n = 17.

CKP1031 Signal peptide – variable region – Constant region

Heavy Chain IgM, amino acid sequence:

MGWSCIILFLVATATGVHS
QVQLQESGPGLAKPSETLSLTCAVSGYSLSSGYYWGWIRQPPGKGLEWIGNIDHSANSYYNPSLKSRVTISVDTSKNEFSLKVRSVTA
ADTAIYYCARDPYSSSSAAFHYWGQGALVTVSS
GSASAPTLFPLVSCENSPSDTSSVAVGCLAQDFLPDSITFSWKYKNNSDISSTRGFPSVLRGGKYAATSQVLLPSKDVMQGTDEHVVC
KVQHPNGNKEKNVPLPVIAELPPKVSVFVPPRDGFFGNPRKSKLICQATGFSPRQIQVSWLREGKQVGSGVTTDQVQAEAKESGPTT
YKVTSTLTIKESDWLGQSMFTCRVDHRGLTFQQNASSMCVPDQDTAIRVFAIPPSFASIFLTKSTKLTCLVTDLTTYDSVTISWTRQNGE
AVKTHTNISESHPNATFSAVGEASICEDDWNSGERFTCTVTHTDLPSPLKQTISRPKGVALHRPDVYLLPPAREQLNLRESATITCLVTG
FSPADVFVQWMQRGQPLSPEKYVTSAPMPEPQAPGRYFAHSILTVSEEEWNTGETYTCVVAHEALPNRVTERTVDKSTGKPTLYNVS
LVMSDTAGTCY

J chain, amino acid sequence:

MGWSCIILFLVATATGVHS
QEDERIVLVDNKCKCARITSRIIRSSEDPNEDIVERNIRIIVPLNNRENISDPTSPLRTRFVYHLSDLCKKCDPTEVELDNQIVTATQSNIC
DEDSATETCYTYDRNKCYTAVVPLVYGGETKMVETALTPDACYPD

Light chain Lamda, amino acid sequence:

MGWSCIILFLVATATGVHS
QTVVTQEPSFSVSPGGTVTLTCGLSSGSVSTNYYPSWYQQTPGQAPRTLIYSTSIRSSGVPDRFSGSILGNKAALTITGAQADDEAHYY
CVLYMGSGISVFGGGTKLTVL
GQPKAAPSVTLFPPSSEELQANKATLVCLISDFYPGAVTVAWKADSSPVKAGVETTTPSKQSNNKYAASSYLSLTPEQWKSHRSYSCQ
VTHEGSTVEKTVAPTECS

CKP1032

Heavy Chain IgM, amino acid sequence:

MGWSCIILFLVATATGVHS
EVQLVESGGGLVQPGGSLRLSCAASGFTFDSYEMNWVRQAPGKGLEWVSYISSSGSTIYYADSVKGRFTISRDNAKNSLYLQMNSLR
AEDTAVYYCARAGEATYYYDSSGYYGLHNMYYFDYWGQGTLVTVSS
GSASAPTLFPLVSCENSPSDTSSVAVGCLAQDFLPDSITFSWKYKNNSDISSTRGFPSVLRGGKYAATSQVLLPSKDVMQGTDEHVVC
KVQHPNGNKEKNVPLPVIAELPPKVSVFVPPRDGFFGNPRKSKLICQATGFSPRQIQVSWLREGKQVGSGVTTDQVQAEAKESGPTTY
KVTSTLTIKESDWLGQSMFTCRVDHRGLTFQQNASSMCVPDQDTAIRVFAIPPSFASIFLTKSTKLTCLVTDLTTYDSVTISWTRQNGEA
VKTHTNISESHPNATFSAVGEASICEDDWNSGERFTCTVTHTDLPSPLKQTISRPKGVALHRPDVYLLPPAREQLNLRESATITCLVTGF
SPADVFVQWMQRGQPLSPEKYVTSAPMPEPQAPGRYFAHSILTVSEEEWNTGETYTCVVAHEALPNRVTERTVDKSTGKPTLYNVSL
VMSDTAGTCY

J chain, amino acid sequence:

MGWSCIILFLVATATGVHS
QEDERIVLVDNKCKCARITSRIIRSSEDPNEDIVERNIRIIVPLNNRENISDPTSPLRTRFVYHLSDLCKKCDPTEVELDNQIVTATQSNICD
EDSATETCYTYDRNKCYTAVVPLVYGGETKMVETALTPDACYP

Light chain Lamda, amino acid sequence:

MGWSCIILFLVATATGVHS
QTVVTQEPSFSVSPGGTVTLTCGLSSGSVSTSYYPSWYQQTPGQAPRTLIYSTNTRSSGVPDRFSGSILGNKAALTITGAQADDESDY
YCVLYMGSGINWVFGGGTKLTVL
GQPKAAPSVTLFPPSSEELQANKATLVCLISDFYPGAVTVAWKADSSPVKAGVETTTPSKQSNNKYAASSYLSLTPEQWKSHRSYSCQ
VTHEGSTVEKTVAPTECS

CKP1033

Heavy Chain IgM, amino acid sequence:

MGWSCIILFLVATATGVHS
QVQLQESGPGLVNPSETLSLSCSVSSVSVSSGNYYWSWIRQPPGKGLEWIGYMSYSGSTTYNPSLKSRVTISLDTSKNQFSLNLNSVT
AADTAVYYCATFATALPGAYFDYWGQGSLVTVSS
GSASAPTLFPLVSCENSPSDTSSVAVGCLAQDFLPDSITFSWKYKNNSDISSTRGFPSVLRGGKYAATSQVLLPSKDVMQGTDEHVVC
KVQHPNGNKEKNVPLPVIAELPPKVSVFVPPRDGFFGNPRKSKLICQATGFSPRQIQVSWLREGKQVGSGVTTDQVQAEAKESGPTTY
KVTSTLTIKESDWLGQSMFTCRVDHRGLTFQQNASSMCVPDQDTAIRVFAIPPSFASIFLTKSTKLTCLVTDLTTYDSVTISWTRQNGEA
VKTHTNISESHPNATFSAVGEASICEDDWNSGERFTCTVTHTDLPSPLKQTISRPKGVALHRPDVYLLPPAREQLNLRESATITCLVTGF
SPADVFVQWMQRGQPLSPEKYVTSAPMPEPQAPGRYFAHSILTVSEEEWNTGETYTCVVAHEALPNRVTERTVDKSTGKPTLYNVSL
VMSDTAGTCY

J chain, amino acid sequence:

MGWSCIILFLVATATGVHS
QEDERIVLVDNKCKCARITSRIIRSSEDPNEDIVERNIRIIVPLNNRENISDPTSPLRTRFVYHLSDLCKKCDPTEVELDNQIVTATQSNICDE
DSATETCYTYDRNKCYTAVVPLVYGGETKMVETALTPDACYPD

Light chain Lamda, amino acid sequence:

MGWSCIILFLVATATGVHS
QTVVTQEPSFSVSPGGTVTLTCGLRSGSVSRNSYPSWYQQMPGQAPRTLIYNTNTRSSGVPHRFSGSILGNKAALTITGAQADDESDY
YCVLYMNDGVVLFGGGTKLTVL
GQPKAAPSVTLFPPSSEELQANKATLVCLISDFYPGAVTVAWKADSSPVKAGVETTTPSKQSNNKYAASSYLSLTPEQWKSHRSYSCQ
VTHEGSTVEKTVAPTECS

Figure S2. Full length sequences used to synthesize the pentameric IgMs CKP1031, CKP1032 and CKP1033. The amino acid sequence for the heavy chain, J chain and light chain are coloured according to the signal peptide (blue), variable region (green) and constant region (black).

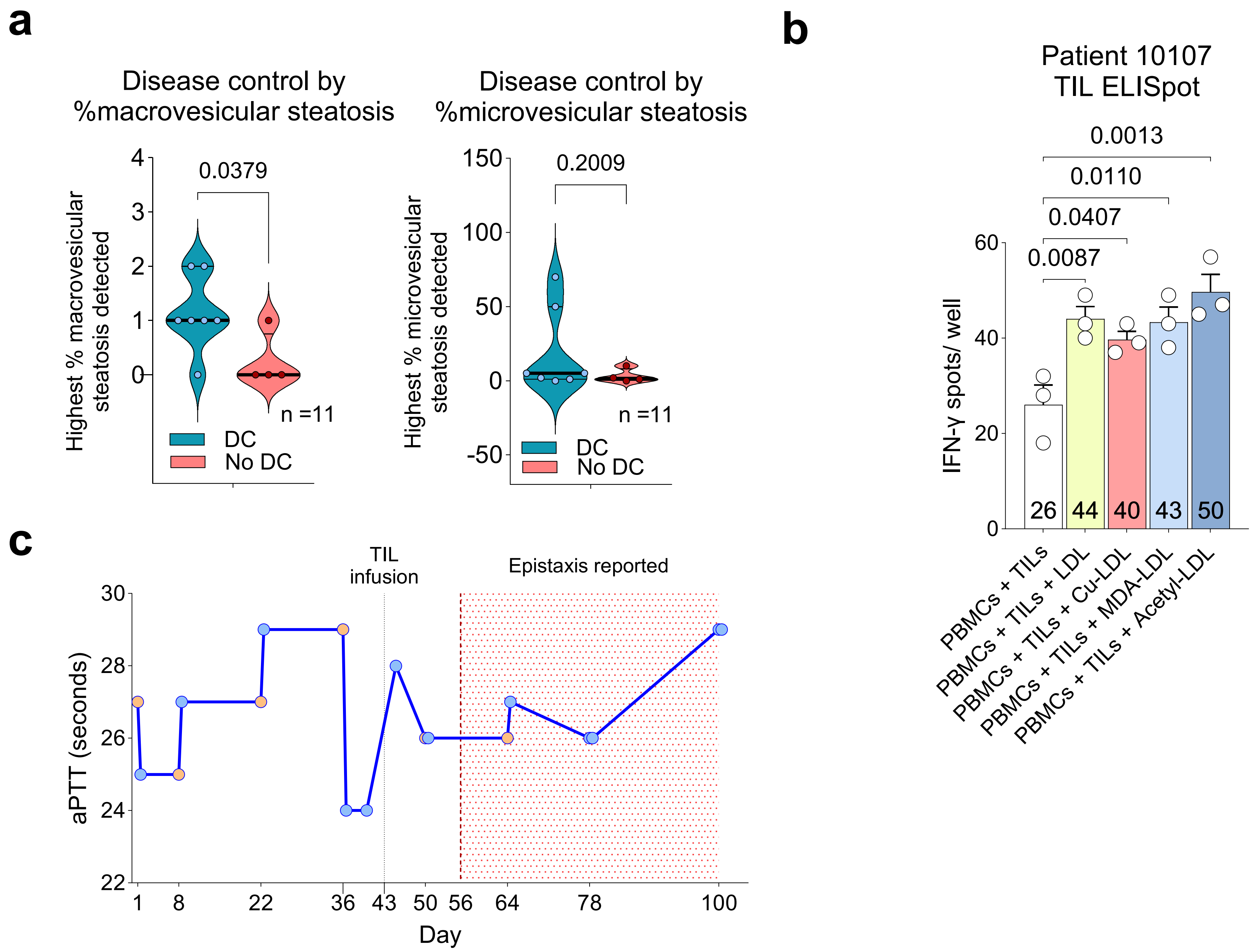

Figure S3. a. Comparison of pathologist-assessed percentages of macrovesicular and microvesicular steatosis in liver biopsies from patients with and without DC. b. Human IFN-γ ELISpot assay assessing the reactivity of the TIL infusion product cultured with PBMCs (baseline) from patient 10107, enrolled in the T215 trial, against native and modified forms (Cu-LDL, MDA-LDL and Acetyl-LDL) of LDL. Mean number of spots is indicated at the bottom of each bar. c. aPTT duration recorded during treatment of patient 10107, with the day of TIL infusion indicated by a black dotted line and the onset of epistaxis marked by a red dotted line. Statistical significance in a. was determined using a two-tailed Mann-Whitney U test. For panel b., significance was assessed using Dunnett's test, with error bars representing the standard error of the mean (SEM).
