# Supplementary material for: Dyslipidemia-associated natural IgM improves oncolytic virus TILT-123 efficacy through antibody-dependent enhancement in solid tumors
Source: Mol Ther. 2026 Jan 20;34(5):3056–76. doi: 10.1016/j.ymthe.2026.01.019 (PMC13154276; doi:10.1016/j.ymthe.2026.01.019)
Supplement: Supplementary file 2 [file mmc3.pdf]

Molecular Therapy
Original Article

Dyslipidemia-associated natural IgM improves oncolytic virus TILT-123 efficacy through antibody-dependent enhancement in solid tumors

James Hugo Armstrong Clubb,[1,2] Santeri Artturi Pakola,[1,13] Sakari Joenväärä,[3,4,13] Tatiana Viktorovna Kudling,[1,2] Tiialotta Tohmola,[3,4] Victor Arias,[1] Elise Jirovec,[1] Mirte van der Heijden,[1] Dafne Carolina Alves Quixabeira,[1,2] Annukka Pasanen,[5] Lyna Haybout,[1,2] Nea Ojala,[1] Saru Basnet,[1] Arianna Eleuteri,[1] Julia Davila Ferrero,[1] Stella Hirvenoja,[1] Inge Marie Svane,[6] Johanna Mäenpää,[7] Katriina Jalkanen,[8] Matthew Stephen Block,[9] Tuomo Alanko,[7] Tine Monberg,[6] Sanae Zahraoui,[2] Susanna Grönberg-Vähä-Koskela,[1,8] Natasha Salmelin,[10] Claudia Kistler,[2] Riikka Havunen,[1,2] Suvi Sorsa,[2] João Manuel dos Santos,[1,2] Victor Cervera-Carrascon,[1,2] Anna Kanerva,[1,11] Otto Hemminki,[1,12] Risto Renkonen,[3,4] and Akseli Hemminki[1,2,8]

[1]Cancer Gene Therapy Group, Translational Immunology Research Program, University of Helsinki, 00290 Helsinki, Finland; [2]TILT Biotherapeutics, 00290 Helsinki, Finland; [3]Transplantation Laboratory, Faculty of Medicine, University of Helsinki, Helsinki, Finland; [4]HUSLAB, Helsinki University Hospital, 00290 Helsinki, Finland; [5]Department of Pathology, University of Helsinki and Helsinki University Hospital, 00290 Helsinki, Finland; [6]National Center for Cancer Immune Therapy (CCIT-DK), Department of Oncology, Copenhagen University Hospital, 2730 Herlev, Denmark; [7]Docrates Cancer Center, 00180 Helsinki, Finland; [8]Comprehensive Cancer Center, Helsinki University Hospital, 00290 Helsinki, Finland; [9]Mayo Clinic Cancer Center, Rochester, MN 55905, USA; [10]IRCM, Insitute de Reserche en Cancérologie de Monpellier, INSERM U1194, University Montpellier, Institute Régional du Cancer Montpellier, Montpellier, France; [11]Department of Obstetrics and Gynecology, Helsinki University Hospital and University of Helsinki, 00290 Helsinki, Finland; [12]Department of Urology, Helsinki University Hospital, 00290 Helsinki, Finland

The oncolytic adenovirus TILT-123 (Ad5/3-E2F-d24-hTNFA-IRES-hIL2, igrelimogene litadenorepvec) has demonstrated favorable safety profiles in phase 1 clinical trials in patients with advanced solid tumors (these trials were registered at ClinicalTrials.gov: NCT04695327, NCT05271318, and NCT04217473). In this study, we analyzed the serum proteome of patients treated with TILT-123 monotherapy using mass spectrometry, focusing on samples collected during the initial intravenous administration phase. Functional and correlative analyses revealed that IGLV8-61-encoded natural immunoglobulin M (IgM) was associated with reduced neutralizing activity and improved clinical outcomes, as assessed by positron emission tomography imaging and overall survival. Single-cell B cell receptor sequencing enabled profiling of the circulating antibody repertoire and detection of IGLV8-61-expressing clones. Recombinant expression of antibody sequences from a responding patient with a history of hyperlipidemia yielded three pentameric natural IgMs capable of binding both TILT-123 and anionic modified low-density lipoprotein. Further characterization revealed that the IgMs significantly enhanced transduction and promoted cell killing *in vitro* across multiple cell lines. Blocking studies demonstrated transduction was influenced by Fc receptors pIgR and FcμR. Cross-trial comparisons indicated dyslipidemia as a common feature among responders. Collectively these findings show that dyslipidemia -associated natural IgM enhances the therapeutic efficacy of TILT-123, via antibody-dependent enhancement. These findings may have broader implications for other oncolytic viruses, an emerging class of tumor immunotherapies.

INTRODUCTION

Oncolytic viruses (OVs) are an immunotherapy used to treat patients with malignant solid tumors. Identifying biomarkers that would aid in the development of strategies to improve intravenous (i.v.) delivery of OVs, will increase the likelihood of successful clinical translation. The oncolytic adenovirus Ad5/3-E2F-d24-hTNFA-IRES-hIL2 (TILT-123) is a next-generation OV designed to augment T cell therapies, including immune checkpoint inhibitors and adoptive cell therapies. TILT-123 was recently evaluated in multiple phase 1 studies across various solid tumor indications, both as a monotherapy and in combination with anti-PD-1 (pembrolizumab) and adoptive cell transfer of tumor-infiltrating lymphocytes (TILs).[1–3] The recent completion of the first phases of the TUNIMO (NCT04695327), PROTA (NCT05271318), and TUNINTIL (NCT04217473) trials demonstrated that TILT-123 is safe and tolerable as i.v., intratumoral (i.t.), or intraperitoneal injections. Durable

Received 14 July 2025; accepted 8 January 2026;
https://doi.org/10.1016/j.ymthe.2026.01.019.

[13]These authors contributed equally

Correspondence: Akseli Hemminki, Cancer Gene Therapy Group, Translational Immunology Research Program, University of Helsinki, 00290 Helsinki, Finland.
E-mail: akseli.hemminki@helsinki.fi

 © 2026 The Author(s).
Published by Elsevier Inc. on behalf of The American Society of Gene and Cell Therapy.
This is an open access article under the CC BY license (http://creativecommons.org/licenses/by/4.0/).

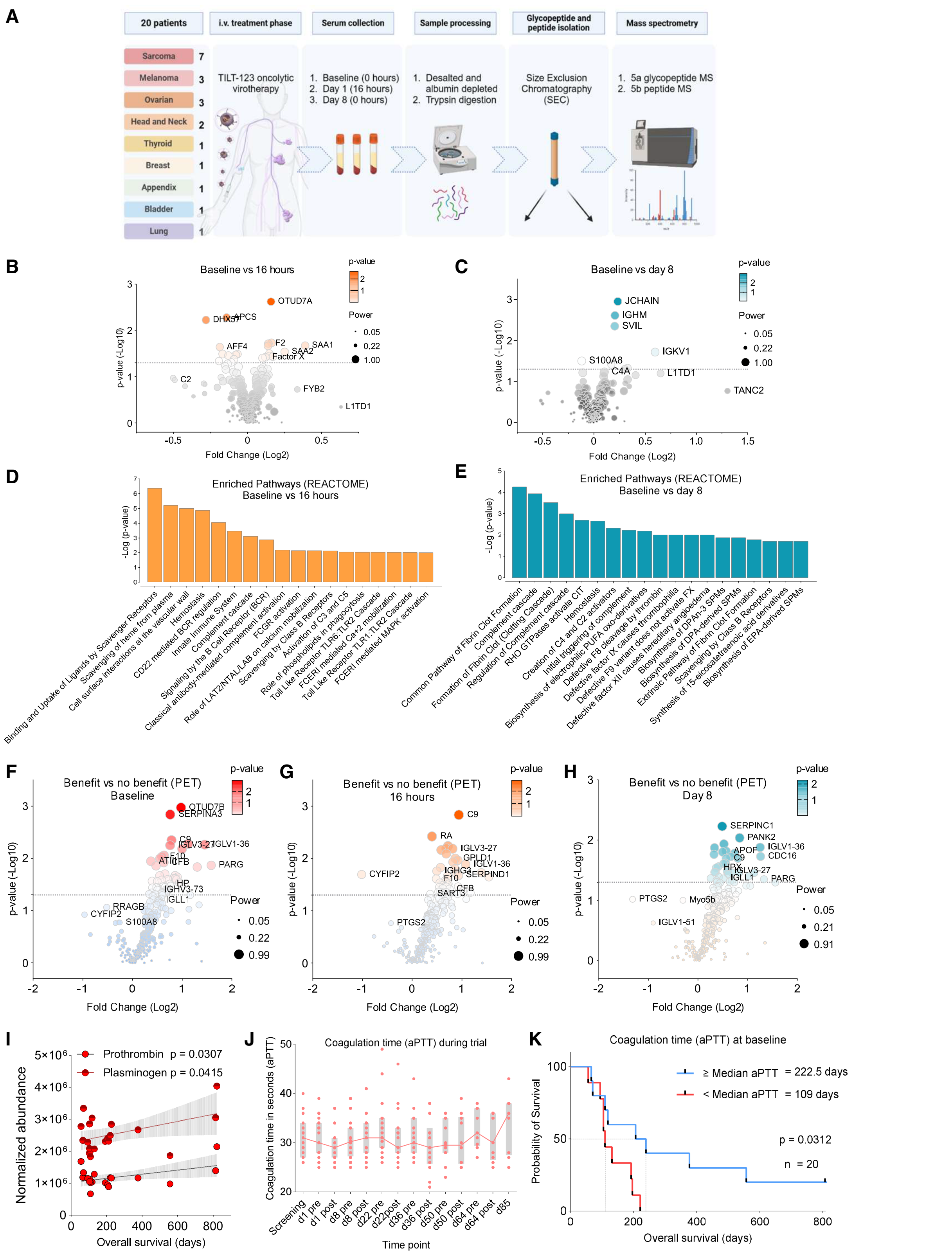

(legend on next page)

responses were observed in difficult-to-treat patients, including those with anaplastic thyroid cancer, mucinous ovarian cancer, and mucosal melanoma.

Patients enrolled in the TUNIMO monotherapy study received an initial i.v. dose, and successful dissemination of TILT-123 from the blood to tumors was observed in some cases.[4] TILT-123 and other virus vectors face significant barriers when delivered systemically, including neutralizing antibodies (nAbs), the complement system, the coagulation system, and clearance by hepatocytes and Kupffer cells in the liver. While nAbs have traditionally been regarded as a barrier to the efficacy of viral vectors, recent findings from our group and others suggest a more nuanced role. Emerging data indicate that nAbs, specifically in the context of hybrid i.v./i.t. delivery, correlate with improved clinical outcomes.[2,5] This observation likely reflects the functional status of patients' B cell immunity, which is increasingly recognized as a critical determinant of antitumor responses in certain cancers, including ovarian cancer. nAbs exist in several forms, each with distinct structural characteristics and mechanisms of action. Among these, natural immunoglobulin M (IgM), a key component of the acute-phase immune response, has been shown to influence the biodistribution and therapeutic efficacy of Ad5-based vectors in murine models, highlighting its potential relevance in determining the clinical activity of OVs.[6] These findings highlight the need to further investigate the role of circulating blood proteins in influencing the pharmacokinetics, pharmacodynamics, and efficacy of OVs such as TILT-123 in the clinical setting.

In this study, we analyzed the proteome of serum collected from patients during the i.v. phase of TILT-123 monotherapy (TUNIMO, NCT04695327). From a responding patient with a history of hypercholesterolemia, we identified three cross-reactive natural IgM antibodies that bind both TILT-123 and anionic forms of modified low-density lipoprotein (LDL), including acetylated LDL (acetyl-LDL) and malondialdehyde-modified oxidized LDL (MDA-oxLDL). A higher abundance of Ig lambda variable 8-61 (IGLV8-61)-associated natural IgM correlated with both a reduction in maximum standardized uptake value (SUVmax) on positron emission tomography (PET) imaging and longer overall survival (OS). Characterization of three IGLV8-61-encoded IgMs isolated from a responding patient revealed that they enhanced *in vitro* transduction and cell killing of cell lines by Ad5/3-luciferase and Ad5/3-d24-E2F, respectively. Transduction was influenced by the polymeric Ig receptor (pIgR) and FcμR (FAIM3), as revealed by a receptor-blocking screen targeting scavenger and Fc receptor candidates.

Cross-trial analysis revealed that a history of dyslipidemia and elevated serum oxidized LDL (oxLDL), particularly 4-hydroxynonenal-modified oxidized LDL (HNE-oxLDL), was associated with favorable clinical outcomes. Notably, the greatest tumor reductions across trials were observed in patients with dyslipidemia and tumors located in regions enriched with natural IgM-producing cells, such as B1 cells and plasma cells within the peritoneal and abdominal cavities. Additional clinical trials with TILT-123 are under way, providing a valuable opportunity to validate these findings in a larger patient cohort.

RESULTS

Patient characteristics and study workflow

We analyzed the serum from patients with different solid tumor types ($n = 20$) treated with TILT-123 monotherapy (TUNIMO trial) using mass spectrometry (MS). The patient characteristics and a schematic representation of the workflow used in this study are summarized in Table S1 and Figure 1A, respectively. Efficacy outcomes from TUNIMO according to response evaluation criteria in solid tumors 1.1 (RECIST 1.1) and PET criteria, as well as median OS are summarized again in Figure S1A. Notably, anaplastic thyroid carcinoma patient 20103 achieved a confirmed partial response, while myxoid liposarcoma patient 20204 achieved an OS of 821 days. The median OS in patients demonstrating disease control (DC) according to PET during the trial was 214 days compared with 109 days in patients with no DC.

Serum samples collected at baseline, 16 h after i.v. injection, and day 8 before the first i.t. injection with TILT-123 were analyzed to identify biomarkers linked to the i.v. phase of treatment. Serum samples were processed by desalting and removing the most abundant protein, albumin. The remaining proteins were then digested using trypsin. Separation of glycopeptide (4a) and peptide (4b) fractions was performed using size-exclusion chromatography before MS analysis as previously described.[7] Analysis of glycopeptides will be reported separately. With the criterion of two or more unique peptides per protein identification, 301 proteins were quantified, and 35 groups were compared using ANOVA with a p value cutoff of 0.05 (Table S1).

Proteomic signature induced by TILT-123

We evaluated changes induced by TILT-123 across time by comparing the proteome at 16 h or at day 8 with baseline using ANOVA. A total of 24 proteins were significantly different 16 h following i.v. injection compared with baseline, 13 of which were

Figure 1. Analysis workflow and TILT-123-induced changes to the serum proteome

(A) Illustration of analysis workflow used in this study (created in BioRender [https://BioRender.com/qfrl8au]). (B and C) Volcano plots illustrating the most significantly upregulated and downregulated proteins at 16 h and 8 days post-treatment compared with baseline. (D and E) Reactome pathway analysis highlighting the most significantly enriched pathways at 16 h and 8 days post-treatment, relative to baseline. (F–H) Volcano plots illustrating the most significantly upregulated and downregulated proteins associated with PET response at baseline, 16 h post-treatment, and on day 8. (I) Correlation of normalized abundance of prothrombin and plasminogen at baseline with OS. (J) Coagulation time as determined by aPTT for all patients in T115 from screening up until day 85. (K) Kaplan-Meier curve showing OS according to patients with greater than or equal to or less than median abundance of aPTT at baseline ($n = 20$). Significance was evaluated using the log rank (Mantel-Cox) test. The color intensity in the volcano plots corresponds to the p value, and the size of the data point corresponds to power. Statistical significance was defined as $p < 0.05$. Source data are provided as Table S1.

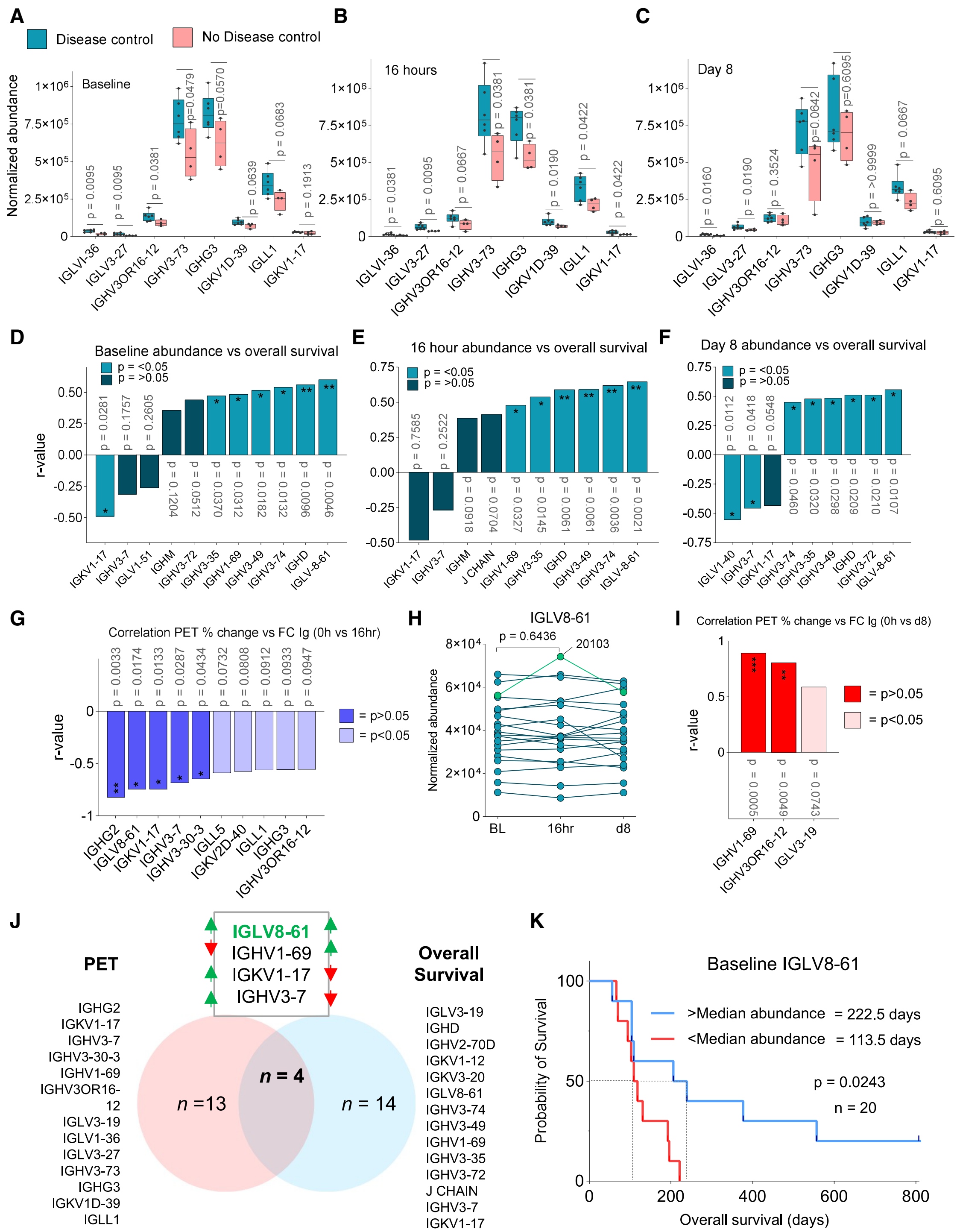

Figure 2. Analysis of serum immunoglobulins identifying IGLV8-61 associated with clinical response

(A–C) Top 8 most consistently significantly different immunoglobulins (Igs) between patients with DC compared to no DC at baseline, 16 h after treatment, and on day 8. (D–F) Most significant correlations between Ig normalized abundance and OS at baseline, 16 h after treatment, and on day 8. Significant correlations and non-significant correlations are colored according to the lighter and dark shade of teal respectively. (G) Most significant correlations between fold change of Igs (16 h vs. baseline) and percentage change in tumor SUVmax (PET). Significant and non-significant correlations are colored according to the darker and lighter shade of blue, respectively. (H) Normalized abundance of IGLV8-61 at baseline, 16 h after treatment, and on day 8 with best-responding patient (20103) highlighted in light green. (I) Summary of most

(legend continued on next page)

upregulated and 11 were downregulated. Proteins involved in the acute phase response (APCS, SAA1, SAA2) and coagulation (F2, SERPINC1, F10) were upregulated, consistent with systemic response to adenovirus infection (Figure 1B). A total of 9 proteins were significantly different 8 days after i.v. injection, 7 of which were upregulated and 2 downregulated. Most upregulated proteins were involved in the acute antibody response (JCHAIN, IGHM, IGKV1-16, IGLV1-40) (Figure 1C).

To gain further insights on proteomic changes induced by i.v. injection of TILT-123 across time, we employed pathway analysis (REACTOME) to identify the most enriched pathways at 16 h and on day 8 (Table S1). We did this by inputting the top 50 most significantly different peptides at each time point compared to baseline. Some of the most enriched pathways 16 h after treatment included binding and uptake of ligands by scavenger receptors, B cell receptor (BCR) signaling, and activation of the complement system (Figure 1D). The most enriched pathways on day 8 included fibrin clot formation, complement cascade, and biosynthesis of long-chain fatty acids (Figure 1E). Overall, the results confirm the accuracy of our analysis because despite the heterogeneous population, we identified pathways involved in the systemic response to adenovirus. These findings prompted us to further investigate proteomic signatures associated with clinical outcomes.

Correlates of clinical outcomes

Preliminary analysis of clinical outcomes was determined by comparing patients with DC against patients with no DC using PET criteria. PET-based criteria have shown greater sensitivity than size-based criteria like RECIST 1.1 and iRECIST in detecting tumor responses to oncolytic virotherapy, where immune-related pseudoprogression is common.[8] The PET criteria are described in Table S2. The PET responses are summarized in Table S3 and Figure S1A.

We then compared the proteome between patients with DC and no DC at baseline, 16 h, and day 8 using ANOVA. At baseline, a total of 45 proteins were significantly more abundant in patients with DC (Figure 1F). At 16 h, a total of 47 proteins were significantly different, with 46 more abundant in patients with DC, and 1 more abundant in patients with no DC (CYFIP2) (Figure 1G). At day 8, 35 proteins were significantly more abundant in DC patients (Figure 1H). PANK2, PLXNA2, ITIH4, IGLV1-36, IGLV3-27, SERPINC1, SERPINA3, SERPIND1, CFB, C9, and F10 were significantly more abundant in patients with DC at all three time points. Again, these were proteins involved in the acute-phase response and those inhibiting the coagulation cascade and clot formation.

Since we aimed to identify a robust marker associated with both DC and OS, we next correlated normalized protein abundance with OS. We initially focused on proteins involved in the coagulation pathway and clot formation, as several anticoagulation proteins were more abundant in patients with DC. A higher normalized abundance of prothrombin ($p = 0.0307$) and plasminogen ($p = 0.0415$) at baseline correlated with longer OS (Figure 1I). To translate these findings, we then evaluated the prognostic value of coagulation rate using activated partial thromboplastin time (aPTT) values collected for safety analysis (Figure 1J). Survival analysis (Kaplan-Meier curve) indicated that patients with a longer baseline coagulation time (greater than or equal to median aPTT) achieved significantly ($p = 0.0312$) longer OS (226 days vs. 109 days) compared with those with a shorter coagulation time (Figure 1K). Preliminary biomarker analysis identified slower coagulation time as a prognostic marker for TILT-123 and highlighted the need to further investigate the prognostic value of other serum proteins.

IGLV8-61 abundance predicts clinical outcomes

Considering that the antibody response influences the bioavailability and efficacy of OV therapy, and our preliminary analysis revealed enrichment of several Ig fragments (hereafter also referred to as Igs) and pathways, we performed analysis of the Ig compartment. Among the 301 proteins quantified by MS, 54 of these were Igs. We first compared normalized abundance between patients with DC and no DC at baseline (Figure 2A), 16 h (Figure 2B), and day 8 (Figure 2C) using the unpaired Mann-Whitney *t* test. At baseline, a higher normalized abundance of IGHV3OR16-12 ($p = 0.0381$), IGHV3-73 ($p = 0.0479$), IGHG3 ($p = 0.0570$), IGKV1D-39 ($p = 0.0639$), and IGLL1 ($p = 0.0683$), in addition to IGLV1-36, IGLV3-27 previously mentioned, were associated with DC. At 16 h, a similar profile was observed with the addition of IGKV1-17 ($p = 0.0422$), while on day 8 only IGLV1-36 ($p = 0.0160$) and IGLV3-27 ($p = 0.0190$) remained significantly associated with DC.

Next, we correlated the normalized abundance of the 54 Igs with OS. At baseline (Figure 2D), six Igs (IGHV3-35, IGHV1-69, IGHV3-49, IGHV3-74, IGHD, and IGLV8-61) significantly correlated with OS, while only one (IGKV1-17) negatively correlated. The same six were consistently significant at 16 h (Figure 2E) and on day 8, with the addition of IGHV3-72 on day 8 (Figure 2F). IGLV1-40 ($p = 0.0112$) and IGHV3-7 ($p = 0.0418$) negatively correlated with OS on day 8. Critically, IGLV8-61 maintained the highest p value and Pearson correlation coefficient at baseline ($p = 0.0046$), 16 h ($p = 0.0021$), and day 8 ($p = 0.0107$). To connect treatment-induced changes in normalized protein abundance to antitumor activity, we correlated fold changes with the percentage change in the SUVmax sum of target lesions for each patient ($n = 10$). Five negative

significant correlations between fold change of Igs (day 8 vs. baseline) and percentage change in tumor SUVmax. Significant and non-significant correlations are colored according to the darker and lighter shades of red, respectively. (J) Venn diagram highlighting Igs associated with PET response and OS. Green and red arrows indicate a positive and negative treatment outcome, respectively.

(K) Kaplan-Meier curve showing OS according to patients with greater than or equal to or less than median abundance of IGLV8-61 at baseline ($n = 20$). Significance was evaluated using log rank (Mantel-Cox) test. Statistical significance was defined as $p < 0.05$.

correlations were significant when correlating fold change from baseline to 16 h, with SUVmax percentage change (Figure 2G). Among these was IGLV8-61 ($p = 0.0174$), indicating that an increase in IGLV8-61 induced by TILT-123 reduced tumor activity. Patient 20103 (partial response) had the highest fold change increase as well as normalized abundance of IGLV8-61 16 h after treatment compared with all other patients (Figure 2H). Only two positive correlations were identified when correlating fold change from baseline to day 8 (Figure 2I). IGHV1-69 ($p = 0.0005$) correlated with an increase in cancer activity despite correlating with longer OS. IGHV1-69 is frequently utilized by broadly nAbs, including those reported against influenza, hepatitis, and SARS-CoV-2.[9–11]

To identify the best Ig candidate to pursue, we compared all significant Ig proteins that were associated with DC by PET and OS (Figure 2J). Only four candidates (IGLV8-61, IGHV1-69, IGKV1-17, and IGHV3-7) were significantly associated with both DC and OS, and only IGLV8-61 was associated with both reduced PET activity (Figure 2G) and longer OS (Figures 2D–2F). We then analyzed the prognostic value of IGLV8-61 and demonstrated that patients with higher than median normalized abundance at baseline achieved almost double OS than those with lower than median (222.5 vs. 113.5 days) (Figure 2K). Altogether, the findings prompted further characterization of IGLV8-61.

IGLV8-61 associated with low neutralizing activity is depleted by systemic therapies

To better understand the Ig response to TILT-123, we first analyzed treatment-induced changes over time using a paired *t* test. IGHV3OR15-7 ($p = 0.0107$), JCHAIN ($p = 0.0006$), IGHM ($p = 0.0010$), IGKV1-16 ($p = 0.0191$), and IGLV1-40 ($p = 0.0441$) were the only five Igs significantly upregulated at either 16 h or day 8 compared with baseline (Figure 3A). Identification of JCHAIN with IGHM together suggested an increase in pentameric IgM aligning with the acute antibody response to virus.[12]

We then sought to identify Igs associated with neutralizing activity against TILT-123. We utilized nAb data from TUNIMO published previously (Figure 3B).[1] Among the 20 patients, 9 had detectable nAbs against Ad5/3 (TILT-123) at baseline (Figure 3C). We compared the pre-treatment proteome between patients with detectable and non-detectable nAbs at baseline. IGHV5-51 ($p = 0.0124$), IGHG1 ($p = 0.0473$), IGKV1-12 ($p = 0.0201$), IGLV3-19 ($p = 0.0437$), IGKV3-7 ($p = 0.0444$) were significantly more abundant in patients with detectable nAbs at baseline (Figure 3D). We then compared the proteome (baseline, 16 h, and day 8) of patients achieving the maximum (high/1:16,384) detectable titer of nAbs after treatment with those that did not reach maximum (low/$\leq$1:4,096). We also compared patients developing a high (>64) and low ($\leq$64) fold change increase in nAb titer from baseline to day 8. IGHV5-51 and IGK1D-33 were significantly more abundant in patients achieving the maximum detectable titer and a high fold change increase, respectively (Figure 3E). In comparison, IGLV8-61 and IGHV1-69 were significantly more abundant in patients developing lower nAb titers and a low fold change increase in nAbs, respectively (Figure 3F). These findings suggest that IGLV8-61 Igs are associated with low neutralizing activity against Ad5/3 and therefore TILT-123.

Next, we aimed to identify an association between IGLV8-61 and clinical characteristics to better define its functional role in treatment benefit. Among the groups included in the ANOVA analysis was comparison of patients with a >5 (high) or $\leq$5 (low) number of previous lines of chemotherapies. CD5 ($p = 0.0481$), IGHM ($p = 0.0186$), and IGLV8-61 ($p = 0.0369$) were significantly more abundant in patients with $\leq$5 previous lines of chemotherapies, while IGKV1D-39 ($p = 0.0063$) and IGKV1-17 ($p = 0.0297$) were significantly more abundant in patients with >5 previous lines of chemotherapies (Figure S1B). Survival analysis comparing the two patient groups revealed a trend ($p = 0.0576$) in longer OS in patients with $\leq$5 previous lines of chemotherapies (131 days vs. 109 days) (Figure S1C). These findings indicate that although chemotherapy appears to deplete IGLV8-61 antibodies associated with treatment benefit, the number of previous treatment lines was not a significant prognostic factor. These findings may parallel observations in rheumatoid arthritis treatment, where B cells are selectively depleted to reduce pathological Ig production. This prompted us to investigate correlations between IGLV8-61 and other serum proteins to explore a potential autoantibody profile.[13,14]

IGLV8-61 correlates with oxLDL

We then correlated normalized abundance of IGLV8-61 with the safety blood values (complete blood count [CBC] with differential) at baseline. aPTT ($p = 0.0486$), hematocrit ($p = 0.0343$), hemoglobin ($p = 0.0425$), alanine transaminase (ALT) ($p = 0.0006$), and creatinine ($p = 0.0343$) significantly correlated with IGLV8-61 (Figure 4A). A correlation between an Ig and prolonged coagulation time suggests natural IgM, which has reported anticoagulant properties through binding to oxidation-specific epitopes (OSEs),[15,16] while elevated ALT and creatinine was consistent with a response to increased immune complexes. Next, we correlated normalized abundance of IGLV8-61 at baseline, 16 h, and day 8 with the respective normalized protein expression (NPX) of serum proteins analyzed with the Olink Target 96 Immuno-Oncology panel and previously reported by our group.[17] At baseline, 13 significant negative correlations were identified, including markers associated with T cell and natural killer cell activation such as CD8A ($p = 0.0025$), GZMA ($p = 0.0179$), KLRD1 ($p = 0.0267$), and ADGRG1 ($p = 0.0419$), and markers associated with myeloid cell migration CXCL1 ($p = 0.0033$), IL-8 ($p = 0.0036$), and CCL23 ($p = 0.0246$) (Figure 4B). A decrease in IL-8 after treatment with oncolytic adenovirus therapy has previously been shown to predict longer OS and better response rates.[18] IL-8 is also known to be produced by monocytes in response to oxLDL.[19] At 16 h, CD40-L ($p = 0.0134$), CXCL1 ($p = 0.0156$), CXCL5 ($p = 0.0156$), and CD8A ($p = 0.0204$) negatively correlated with IGLV8-61 (Figure S1D). On day 8, there were 9 significant negative correlations, including IL-5 ($p = 0.0029$), KIR3DL1 ($p = 0.0237$), PTN ($p = 0.0328$), TNFARSF12A ($p = 0.0438$), and TNFASF14 ($p = 0.0430$), and 1 significant positive correlation with

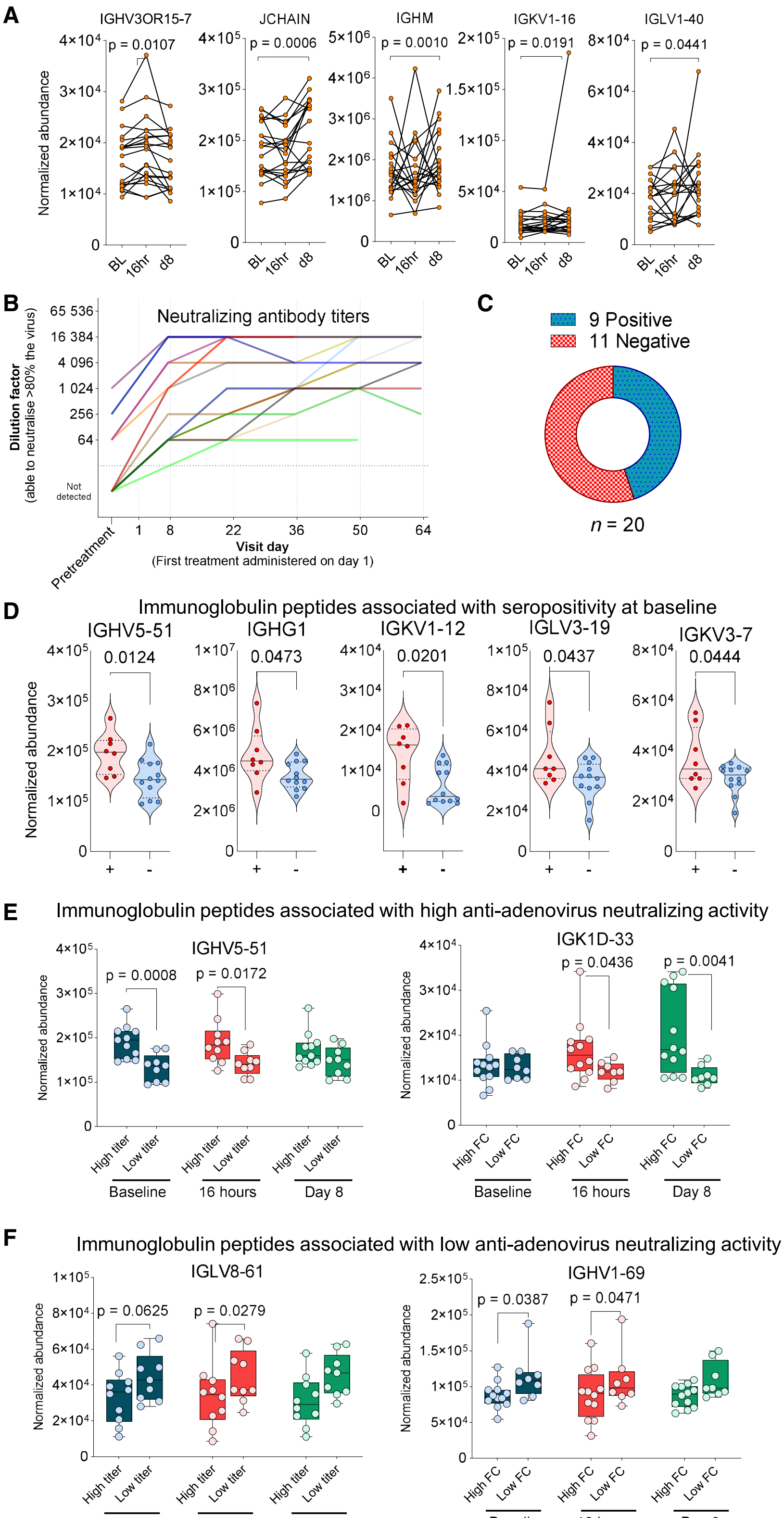

Figure 3. IGLV8-61 is associated with low neutralizing activity against TILT-123

(A) Normalized abundance of five Igs exhibiting statistically significant changes over time. (B) Summary of nAb titers for all patients enrolled in T115 during the trial. (C) Summary of number of patients in T115 with detectable and non-detectable titers of nAbs at baseline. (D) Group comparison of normalized Ig abundance between patients with detectable (≥1:64) and non-detectable (<64) baseline nAb titers, highlighting the five most significantly different Igs. (E) Group analysis identifying the Igs most significantly associated with neutralizing activity at baseline, 16 h, and day 8, based on baseline nAb detectability and treatment-induced fold changes. (F) Group analysis identifying Igs most significantly associated with low neutralizing activity at baseline, 16 h, and day 8, based on baseline nAb detectability and treatment-induced fold changes. Significance in (A) was calculated using paired *t* test, while comparison between groups was evaluated by two-tailed Mann-Whitney *U* test. Statistical significance was defined as $p < 0.05$. FC, fold change; high FC, >64 FC increase; high titer, ≥1:1,024; low titer, ≤1:256; low FC, <64 FC increase.

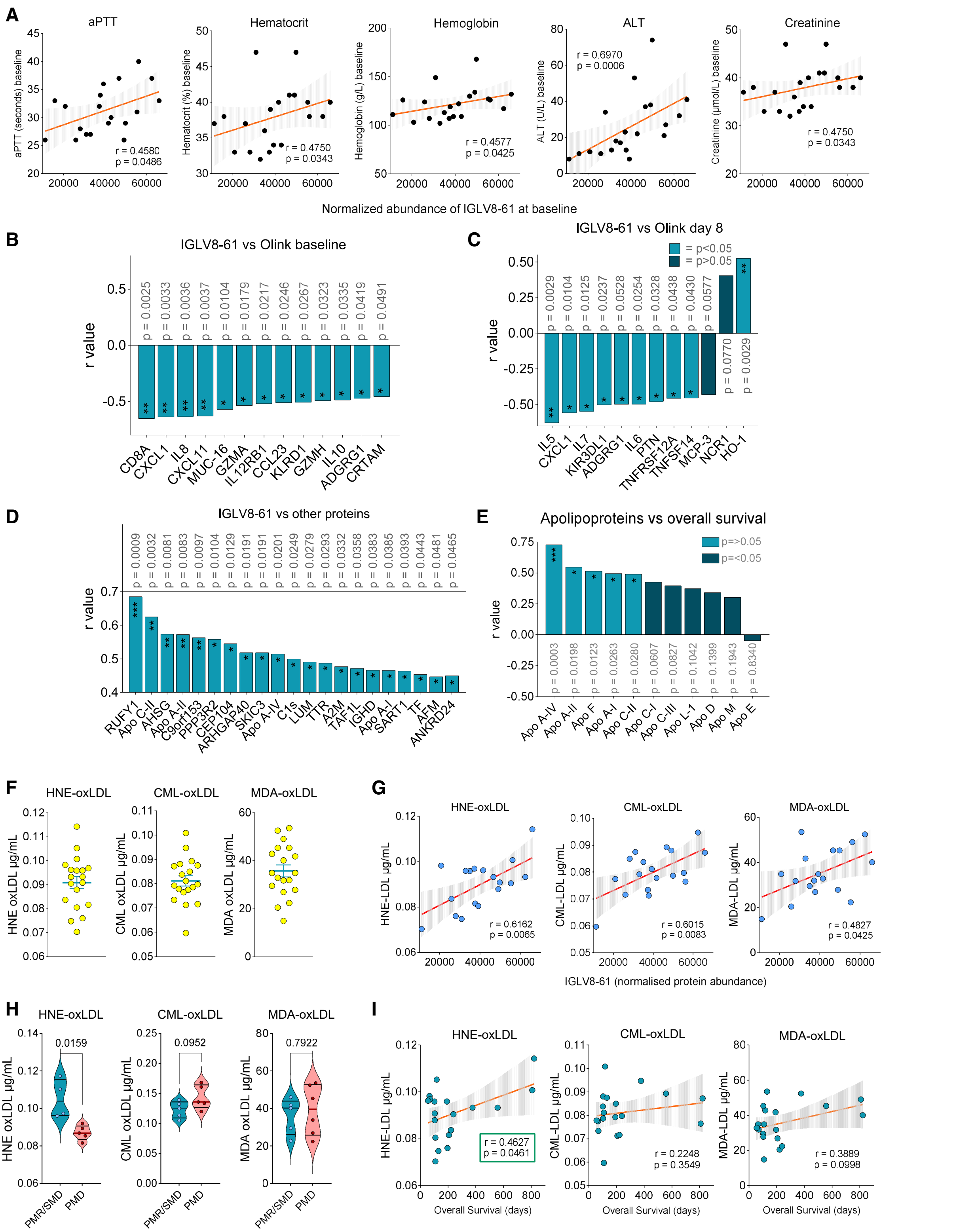

(legend on next page)

HO-1 (p = 0.0029; Figure 4C). IL-5 has known roles in innate B1 cell expansion and production of natural IgM against OSE,[20] while HO-1 is elevated in response to oxidative stress, including oxLDL.[21,22] We also correlated normalized abundance of IGLV8-61 with the 300 proteins detected by MS at baseline (Figure 4D). We identified 21 significant positive correlations, including RUFY1 (p = 0.0009), AHSG (p = 0.0081), A2M (p = 0.032), AFM (p = 0.0481), and apolipoproteins Apo-CII (p = 0.0032), Apo A-II (p = 0.0081), Apo A-IV (p = 0.0201), and Apo A-I (p = 0.0385). A higher abundance of all four apolipoproteins in addition to Apo F significantly correlated with longer OS (Figure 4E).

These findings prompted us to quantify serum oxLDL and correlate oxLDL concentration with IGLV8-61 and clinical outcomes. The analysis of oxLDL included three commercially available kits that detect HNE, MDA, and carboxymethyl lysine (CML)-modified oxLDL (Figure 4F). HNE-oxLDL (p = 0.0065), MDA-oxLDL (p = 0.0083), and CML-oxLDL (p = 0.0425) significantly correlated with normalized abundance of IGLV8-61 at baseline (Figure 4G). Analysis of concentration according to DC or no DC revealed that only HNE-oxLDL was significantly (p = 0.0159) associated with DC compared with CML and MDA-oxLDL (Figure 4H). Similarly, only HNE-oxLDL significantly (p = 0.0461) correlated with longer OS (Figure 4I). Overall, these findings suggest that IGLV8-61-associated antibodies are natural IgMs produced in response to elevated levels of modified LDL and that a higher abundance of specifically HNE-modified oxLDL is more strongly associated with favorable clinical outcomes.

IGLV8-61 natural IgM binds TILT-123 and anionic modified LDL

To validate our assumptions, we performed flow cytometry to phenotype circulating natural IgM-producing cells, including IgM^+ B1 B cells and plasma cells, and correlate their abundance with clinical outcomes. The percentage of $CD3^-$, $CD19^+$, $CD27^+$, $CD43^+$, and $CD5^+$ B1a B cells out of the total cells across all patients ranged from 0.052% to 1.58% and did not appear to be associated with imaging response by PET (Figure S1E). However, a higher percentage of B1a B cells correlated with longer OS on both baseline and on day 8 (Figure S1F). We also analyzed the percentage of circulating IgM^+ $CD38^+$ plasma cells and found a stronger, statistically significant positive correlation with OS compared to B1a B cells, both at baseline and on day 8 (Figure S1G).

We then utilized single-cell BCR sequencing of peripheral blood mononuclear cells (PBMCs) previously generated by our group to identify the full-length sequence of IGLV8-61-containing clones.[23] Full-length BCR data were available for six patients (20103, 20108, 20211, 20212, 20217, and 20219) at baseline and day 64 of treatment. The total number of Igs expressing cells detected according to patient, time point, and isotype are summarized in Figure 5A. IGHM was the most abundantly detected Ig isotype. Among the 2022 identified B cell clones, 16 utilized the IGLV8-61 light chain, and notably, 9 of these 16 also expressed the IGHM heavy chain (Table S4). This pattern supports the presence of natural IgM, which is typically characterized by germline-encoded, low-affinity antibodies produced by innate-like B cells.

We subsequently synthesized three pentameric IGLV8-61 IgM clones derived from the responding patient 20103 (partial response) to evaluate their binding properties. These antibodies were tested against TILT-123 and various commercially available forms of LDL, including native LDL, acetyl-LDL, and oxLDL (MDA-oxLDL and copper oxLDL [Cu-oxLDL]). The selected clones, number 2 (CKP1031), number 3 (CKP1032), and number 4 (CKP1033), were reconstructed from single B cell clones identified from PBMCs collected on day 64 of treatment (Figures 5B and S2; Table S4). We performed indirect ELISAs to assess the binding of each clone to TILT123 (Figure 5C) and the various forms of LDL (Figure 5D). All three clones bound TILT-123 in a dose-dependent manner (Figure 5E). We then compared their ability to bind native LDL and its modified forms. All three clones—CPK1031 (p = 0.0009), CKP1032 (p = 0.0474), and CPK1033 (p = 0.0034)—exhibited significantly greater binding to acetyl-LDL compared with native LDL (Figure 5F). Similarly, all clones showed significant binding to MDA-oxLDL compared with native LDL (Figure 5G). In contrast, no significant difference in binding was observed with Cu-oxLDL and native LDL (Figure S1H). These data demonstrate that the three IGLV8-61-encoded natural IgM clones, isolated from a responding patient with a history of hypercholesterolemia, bind both TILT-123 and modified forms of LDL, with increasing affinity corresponding to the degree of negative charge on the LDL particles.

IGLV8-61 IgM augments adenoviral transduction and cytotoxicity *in vitro*

To investigate how CPK1031, CPK1032, and CPK1033 enhance the efficacy of TILT-123 and whether this effect involves scavenger or Fc receptor pathways, we conducted *in vitro* experiments assessing the IgM-Ad5/3 complex in transduction, cell viability, and receptor-blocking assays. As an initial step, we evaluated the impact of

Figure 4. IGLV8-61 is associated with clinical characteristics, immune cell markers, and abundance of oxLDL

(A) Significant correlations between baseline abundance of IGLV8-61 and clinical parameters at baseline, including aPTT, hematocrit, hemoglobin, ALT, and creatinine. (B and C) Significant correlations between IGLV8-61 abundance at baseline and day 8, with the corresponding normalized protein expression values of immuno-oncology-related proteins, as measured by Olink analysis. (D) Significant correlations between baseline IGLV8-61 abundance and all other proteins identified by MS. (E) Correlations between baseline abundance of all apolipoproteins detected by MS and OS. (F) Quantification of serum concentrations of HNE-oxLDL, CML-oxLDL, and MDA-oxLDL in all T115 (cohort 1–6) patients at baseline. (G) Correlations between baseline abundance of IGLV8-61 with HNE-oxLDL, CML-oxLDL, and MDA-oxLDL. (H) Comparison of baseline HNE, CML, and MDA modified oxLDL concentrations between patients with and without DC, as defined by PET criteria. (I) Correlations between baseline serum concentrations of HNE-oxLDL, CML-oxLDL, and MDA-oxLDL with OS. In (B)–(E), significant and non-significant correlations are indicated by the lighter and darker shades of teal, respectively. Statistical significance between groups was assessed using a two-tailed Mann-Whitney U test. DC, disease control. Statistical significance was defined as $p < 0.05$.

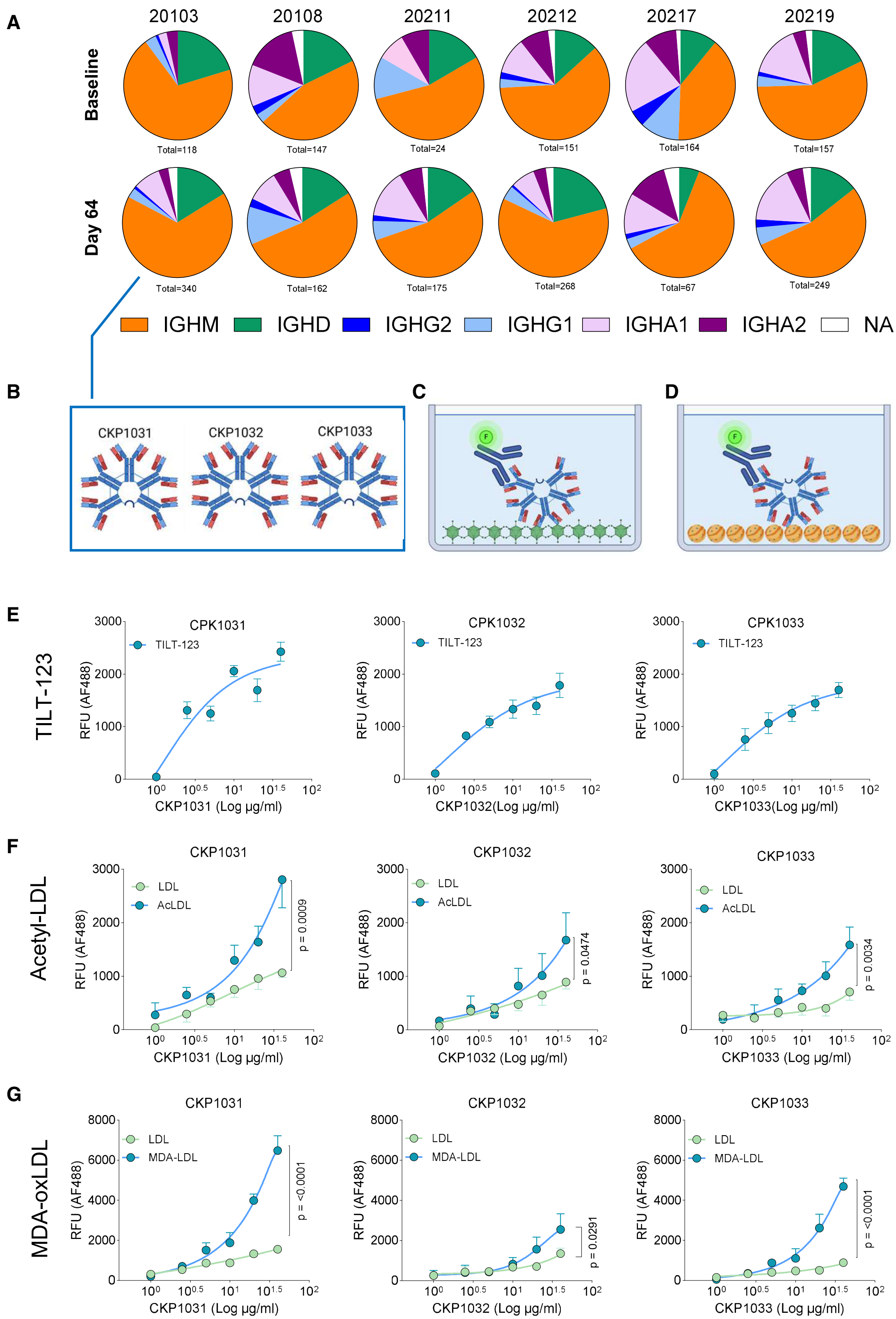

(legend on next page)

each IgM clone as a monotherapy on cancer cell viability. A dose-response assay was performed on A549, SW620, and SCOV3 cell lines (Figure 6A). All three IgM clones significantly increased cell viability in all cell lines after 24 h, except at the highest concentration (50 μg/mL), where viability began to either decline or plateau. These findings indicate a trophic role for IgM uptake in supporting the growth and survival of these cancer cell lines.

We next assessed the impact of IgM clones on viral transduction using a luciferase-expressing, non-replicative Ad5/3 vector (Ad5/3-Luc) (Figure 6B). The vector was pre-complexed at 10, 100, or 1,000 virus particles per cell (VP/cell), with increasing concentrations of CPK1031, CPK1032, or CPK1033 prior to overnight incubation with A549, SW620, and SCOV3 cells. Luciferase assays demonstrated a dose-dependent enhancement of transduction across all cell lines, with the most consistent effect observed at 100 VP/cell. To determine whether enhanced transduction was associated with increased cytotoxicity, we performed dose-response experiments using Ad5/3-d24-E2F (TILT-123 backbone) and assessed cell viability with the MTS assay, focusing on OV-mediated oncolysis (Figure 6C). By day 3, all three IgM clones significantly increased cell killing in A549, SW620, and SCOV3 cells in a concentration-dependent manner.

To identify potential receptors mediating the observed enhancement in transduction, we screened a panel of antibodies targeting key scavenger and Fc receptors, including pIgR, FAIM3, CD351, CD206, LOX1, and CD36, using A549 cells (Figure 6D). Blocking studies were performed with CPK1033 and Ad5/3-Luc at 100 VP/cell, based on findings from Figures 6B and 6C. No notable differences were observed between the standard treatment (Ad5/3-Luc + IgM) and cells pre-treated with antibodies against CD351, CD206, LOX1, CD36, or their respective isotype controls. However, unexpectedly, pre-treatment with antibodies targeting pIgR or FAIM3, two receptors known to mediate IgM immune complex binding and uptake, resulted in an increase in Ad5/3-Luc transduction.

Given that some cancer types actively downregulate pIgR and that FAIM3 expression in cancer remains poorly characterized, we next sought to confirm protein expression of both receptors to ensure that these observations were biologically plausible (Figure 6E). Using immunohistochemistry (IHC) on A549 spheroids, we confirmed expression of both pIgR and FAIM3, supporting their potential role in influencing the uptake of Ad5/3-IgM immune complexes in this context. Additionally, we observed a significant positive correlation between the abundance of IGLV8-61 and RUFY1, a key regulator of endosomal recycling, suggesting a possible link between IgM-mediated viral uptake and intracellular trafficking pathways (Figure 4D).

These findings demonstrate that IGLV8-61 IgM promotes the proliferation of cancer cell lines and enhances Ad5/3 viral transduction and oncolytic activity *in vitro*. The observed effects appear to be negatively regulated by pIgR and FAIM3 in A549 cells.

Dyslipidemia correlates with clinical outcomes across trials

To validate our findings of dyslipidemia-associated improved clinical outcomes, we performed cross-trial analyses of HNE-oxLDL, CML-oxLDL, and MDA-oxLDL using samples collected from TUNIMO (T115) cohort 7, PROTA (T563-1a), and TUNINTIL (T215) (Figure S1I). In total there were 6, 15, and 17 patients in T115 cohort 7, T563-1a, and T215, respectively. The treatment schedules for each trial are illustrated in Figure 7A. We correlated baseline concentrations of oxLDL with OS and found that HNE-oxLDL positively correlated with longer OS in TUNIMO cohort 7 (p = 0.0436) and T563-1a (p = 0.0010) but not T215 (p = 0.8390) (Figure 7B). Of note, patient 10107 (pathological complete response) from T215 was among the outliers, exhibiting one of the lowest concentrations of oxLDL. Correlations of CML-oxLDL and MDA-oxLDL revealed a similar finding, as previously observed, with neither significantly correlating with OS in T563-1a and T215. However, there was a nearly significant (p = 0.0557) negative correlation of CML-oxLDL with OS in T215 (Figures S1J and S1K). Conversely, CML-oxLDL (p = 0.0431) and MDA-oxLDL (p = 0.0583) both showed a significant positive correlation with longer OS in TUNIMO cohort 7. These findings support the previous observation we made with T115 cohort 1–6 that HNE-oxLDL is particularly beneficial for positive clinical outcomes.

Survival analysis (Kaplan-Meier curves) confirmed the prognostic value of HNE-oxLDL. Patients in TUNIMO with baseline HNE-oxLDL values higher than the median achieved (p = 0.0163) an OS of 258 days, compared with 129 days for patients with values below the median (Figure 7C). Similarly, patients in T563-1a with baseline HNE-oxLDL values higher than the median (p = 0.0122) achieved an OS of 340 days, compared with 138 days (Figure 7D). Although not statistically significant, patients in T215 with higher values of HNE-oxLDL achieved an OS of 625 days (p = 0.2971), compared with 369 days for those with values below the median (Figure 7E). These findings prompted us to compare the best responses between all

Figure 5. Identification of three natural IgM clones binding both TILT-123 and modified LDL

(A) Summary of single-cell analysis of circulating antibody-producing cells in six patients, showing the number of identified single cells per isotype at baseline and day 64. (B) Illustration of three individual IGLV8-61-expressing clones (CKP1031, CKP1032, CKP1033) isolated from patient 20103 on day 64 (created in BioRender [https://BioRender.com/7ik9fd1]). (C) Illustration of the indirect ELISA setup used to assess the binding of IgM clones to TILT-123 (created in BioRender [https://BioRender.com/51c3ndq]). (D) Illustration of the indirect ELISA setup used to assess the binding of IgM clones to LDL and modified LDL (created in BioRender [https://BioRender.com/cweb5tq]). (E) Binding analysis of IgM clones to TILT-123, demonstrating a dose-dependent increase in fluorescence signal (Alexa Fluor 488) with increasing concentrations of each clone. (F and G) Fluorescence-based binding assay demonstrating increased binding of IgM clones to acetylated LDL and MDA-modified oxLDL compared to native LDL, as indicated by significantly higher fluorescence signal intensity. Significance was calculated using two-way ANOVA mixed-effects analysis. Statistical significance was defined as $p < 0.05$.

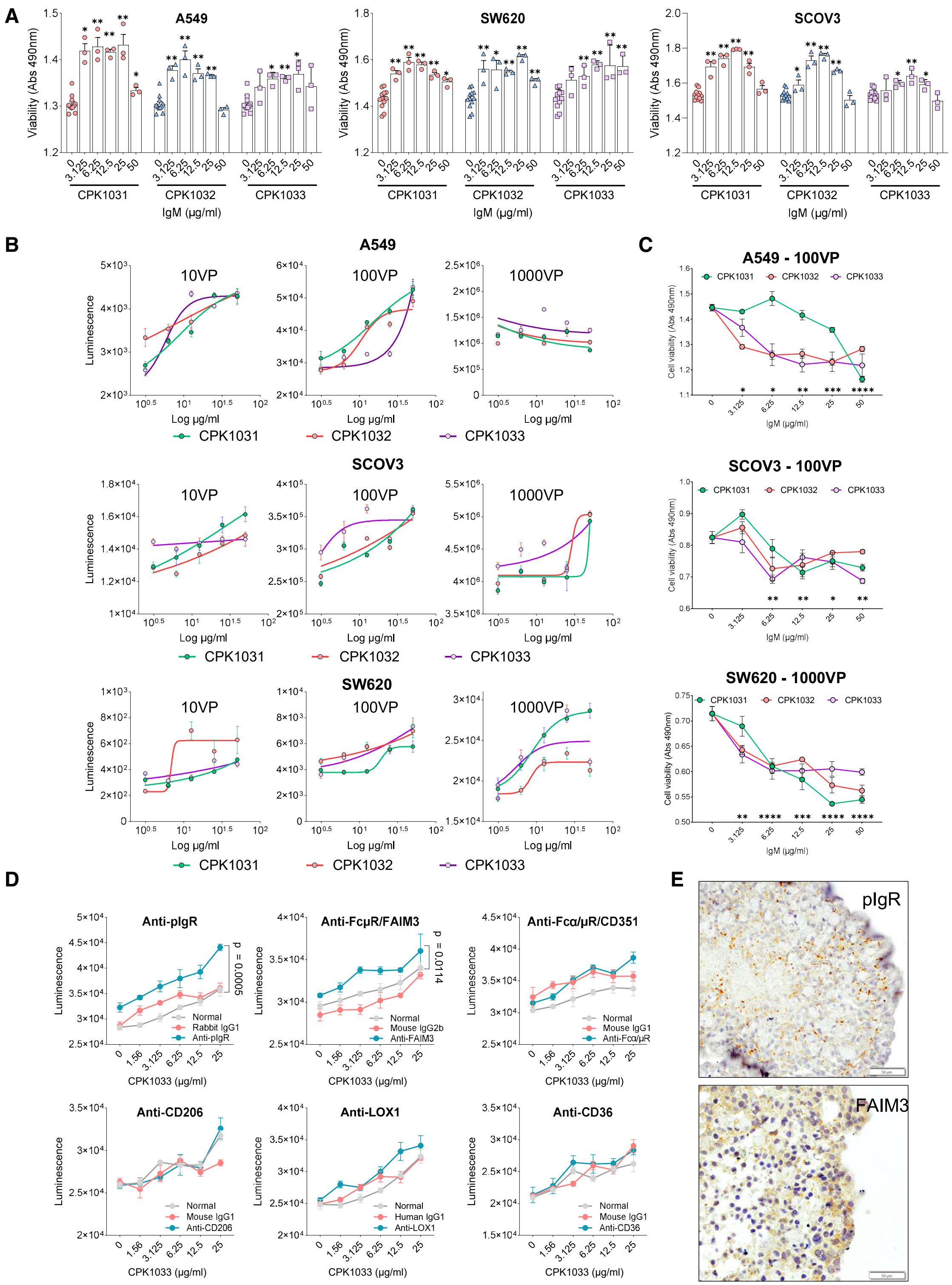

(legend on next page)

patients in T115 and T563-1a and those with a history of hyperlipidemia in their patient records. For all patients, the overall response rate (ORR) and the DC rate (DCR) were 9.7% and 41.5%, respectively (Figure 7F). In contrast, the ORR and DCR for patients with a history of hyperlipidemia were 25% and 100%, respectively (Figure 7G). We also found that patients with a history of hyperlipidemia in T115 and T563-1a achieved a median OS of 327 days, compared with 127 days in all other patients (Figure 7H).

Given that dyslipidemia is known to impact the liver, including hepatocyte and Kupffer cell-mediated scavenger function, we conducted a cross-trial analysis of available liver biopsy samples to provide insight into liver status. We quantified the percentage of macrovesicular and microvesicular steatosis in liver biopsies collected from patients T115, T563-1a, and T215, with assessments performed by a pathologist. A higher percentage of macrovesicular steatosis was significantly (p = 0.0379) associated with patients achieving DC, whereas microvesicular steatosis was not (p = 0.2009) (Figure S3A). We also examined patient outlier 10107 to understand the possible cause of the anomaly and to potentially gain a better understanding of the relation between dyslipidemia and clinical benefit. Preliminary T cell receptor (TCR) binding predictions on the TIL infusion product using TCRmatch, conducted as part of a previously published case report, identified the most expanded T cell clone—predominantly $CD4^+$ T cells—as sharing over 90% sequence similarity with a known TCR specific for APOB/apolipoprotein B-100 (apoB-100). Notably, apoB-100 is a major structural component of LDL and its oxidized forms.[24] To validate this finding, we performed an interferon-γ (IFN-γ) ELISpot assay by co-culturing patient-matched PBMCs collected at baseline with the TIL infusion product in the presence of native LDL and modified forms of LDL. We observed significantly more spots in conditions cultured with both native LDL and modified forms (Cu-oxLDL, MDA-oxLDL, and acetyl-LDL) compared with the control (Figure S3B). The patient also experienced intermittent epistaxis before and after TIL infusion, including a period reported 13 days after TIL infusion (Figure S3C).

Lastly, we analyzed the three best-responding patients in cohorts T215, T115 phase 1a (cohorts 1–6), and T563 phase 1a to identify shared clinical features. All three exhibited dyslipidemia, either apoB-100 autoimmunity or hyperlipidemia, and had undergone relatively few prior chemotherapy treatments. Notably, the two patients with hyperlipidemia had tumors locally injected with TILT-123 in the peritoneal or abdominal cavity (Figure 7I). We therefore performed cross-trial (T115 and T563-1a) analysis of tumor size changes by computed tomography (CT) imaging and identified that abdominal/peritoneal tumors responded better (p = 0.0127) to TILT-123 than tumors in other anatomical locations (Figure 7J), a finding observed in previous clinical experience with oncolytic adenovirus therapy.[25]

DISCUSSION

Although durable responders have been observed, efforts to establish oncolytic virotherapy in clinical practice so far have been less successful than immune checkpoint inhibitors, primarily due to lower response rates. This is also partly due to the current unpredictability of responses, which stems from the challenge of identifying robust biomarkers in multimodal therapies such as OVs. In principle, OVs should be able to replicate and lyse a broad range of solid tumor types, as demonstrated by our group and others through consistent lysis of diverse cancer cell lines *in vitro* and *in vivo*. This suggests that in clinical practice, one of the most crucial factors for therapy success is effective delivery of the virus to tumor sites and the initiation of i.t. replication. This is particularly relevant as developers of OVs transition to i.v.-only delivery approaches to improve clinical feasibility, patient accessibility, and perhaps better delivery to metastases, including micrometastases. Preliminary biomarker analyses by our group support this observation, as longer OS was observed in patients with a detectable viral presence in tumor biopsies following a single i.v. dose of TILT-123.[4] For OVs to achieve tumor transduction, oncolysis, and an immune response, they must overcome antiviral immunity, including complement, coagulation, and nAbs. In this work, we report the identification of IGLV8-61-encoded natural IgM and dyslipidemia, particularly elevated HNE oxLDL as a potential biomarker for TILT-123. Moreover, using single-cell BCR sequencing, we identified three IGLV8-61-encoded natural IgM clones from a responding patient with hyperlipidemia and demonstrated their cross-reactivity with both TILT-123 and modified anionic forms of LDL (MDA- and acetyl-LDL) *in vitro*. Further characterization demonstrated that these IgMs enhance both Ad5/3 transduction and cancer cell killing, an effect subsequently linked to Fc receptor involvement, specifically pIgR and FAIM3. Although this suggests a novel mechanism, the precise endocytic pathways and specific receptors mediating this enhanced efficacy remain to be fully defined. While IgM binding provides an alternative cellular entry pathway, the observed significant positive correlation between IGVL8-61 abundance and RUFY1 suggests a critical secondary role in prolonged early endosomal maturation. This delay could facilitate improved adenoviral endosomal escape by reducing lysosomal degradation, thereby enhancing virus replication. Further mechanistic studies are required to confirm these hypothesized alterations in endocytic trafficking.

Most natural IgM are polyreactive antibodies encoded by germline Ig sequences and secreted by B1 B cells and plasma cells that reside

Figure 6. IGLV8-61 natural IgM clones promote Ad5/3 transduction and cell killing *in vitro*

(A) Viability analysis of A549, SW620, and SCOV3 cells treated with CPK1031, CPK1032, and CPK1033 monotherapy. (B) Dose-response curves demonstrating the effect of increasing concentrations of these clones on Ad5/3-luciferase transduction at 10, 100, and 1,000 VP/cell. (C) Dose-response curves illustrating their impact on Ad5/3-d24-E2F-mediated cell viability. (D) Blocking screen of six candidate receptors (pIgR, FAIM3, CD351, CD206, LOX-1, and CD36) or isotype controls for Ad5/3-Luc transduction at 100 VP/cell in A549 cells with CPK1033, including immunohistochemistry detection of pIgR and FAIM3 in A549 spheroids, with statistical significance assessed by paired t test in (A) and (B) versus 0 μg/mL IgM (*$p < 0.05$; **$p < 0.01$; *$p < 0.001$) and by two-way ANOVA mixed-effects analysis in (D). Statistical significance was defined as $p < 0.05$.

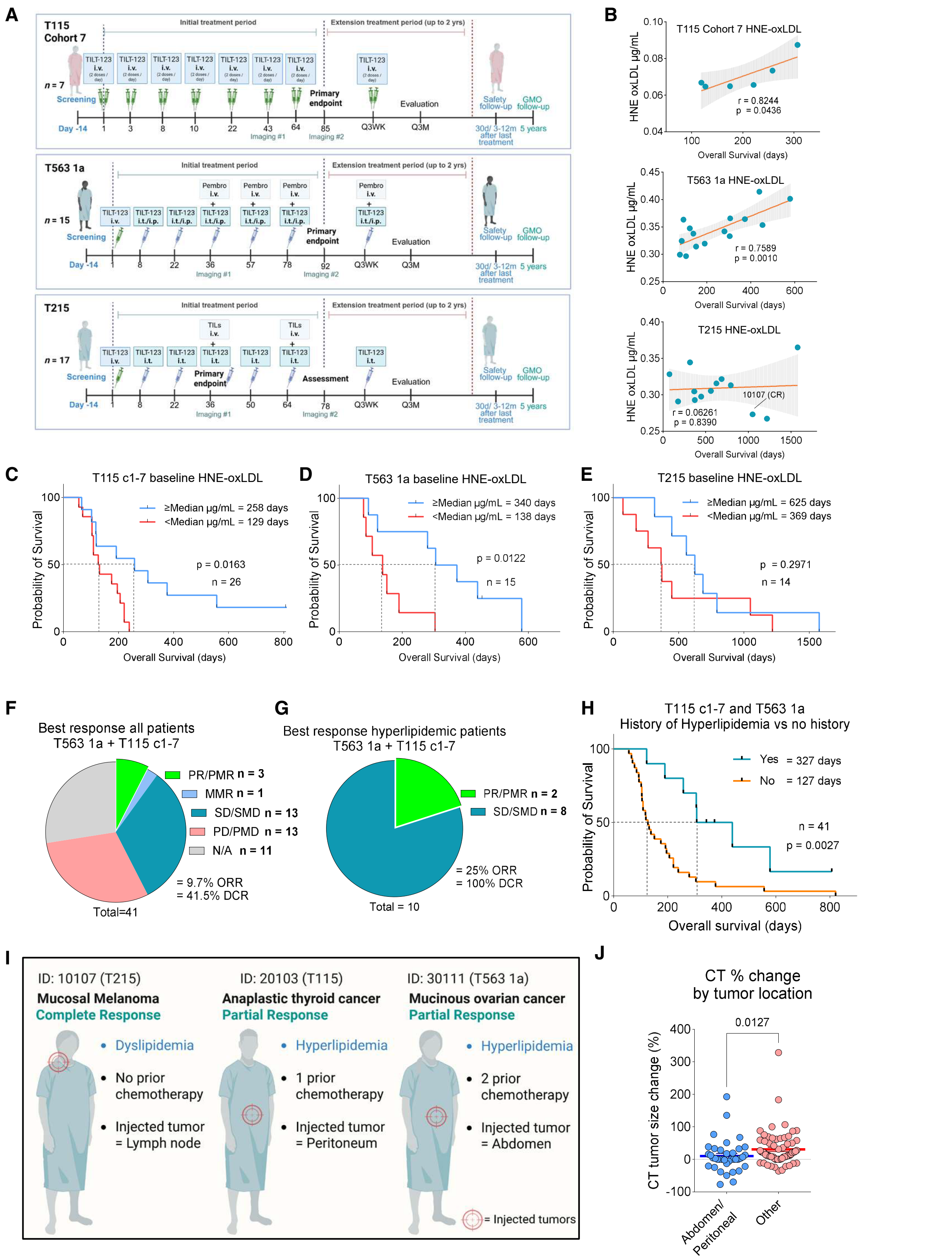

(legend on next page)

primarily in the peritoneum and pleural cavities.[26] Their key functions include protection against viral, bacterial, and fungal infections, as well as promotion of the clearance of apoptotic cells, necrotic cells, and modified LDL. The role of natural IgM in adenovirus gene therapy has been reported in mice, primarily indicating a negative impact on bioavailability, by increasing clearance through Kupffer cells.[27] In comparison, dyslipidemia-associated IGLV8-61 IgM was found to enhance treatment efficacy by promoting adenoviral transduction and oncolytic cytotoxicity, consistent with a mechanism resembling antibody-dependent enhancement (ADE).

ADE of viral infections typically requires binding of the virus to sub-optimal nAbs, followed by uptake via phagocytic Fc receptors or complement receptors. As a result of sub-optimal neutralization, this eventually leads to increased viral infectivity, virulence, and persistence, as observed in this study. This is supported by prior studies showing that natural IgM against adenovirus typically possesses minimal neutralizing capacity *in vitro*; accordingly, the presence of IGLV8-61 was found to correlate with low nAb titers both pre-treatment and following TILT-123 administration.[28,29] Analysis of TILT-123-specific DNA present in tumor biopsies collected during our trials further supports our observations of ADE, specifically virus persistence. Among the available tumor biopsies in the TUNIMO trial (T115), three patients (20204, 20206, and 20212) demonstrated viral positivity by qPCR. Remarkably, this included biopsies collected 8, 14, and even up to 20 days after the last prior treatment with TILT-123, with the latter even being a non-injected tumor in patient 20204. Interestingly, all positive biopsies were collected from tumors located in the peritoneum or abdominal wall. This observation of viral persistence may explain our observation of superior tumor responses in these anatomical locations, as observed in this and previous studies.[25] Furthermore, all three patients exhibited relatively high serum oxLDL concentrations, with patient 20204 demonstrating the highest level. Notably, this patient, who had type 2 diabetes (a condition strongly associated with elevated oxLDL), also achieved the second-longest OS in the T115 trial. The influence of other comorbidities and associated medication will be the focus of future research. A similar observation was made in the PROTA ovarian cancer trial (T563-1a). Again, three patients (30206, 30210, and 30111) demonstrated viral positivity in biopsies by qPCR, all had a history of hyperlipidemia, and all had tumors in the peritoneum, abdominal wall, or spleen. Similarly, this included tumors biopsied up to 20 days after the last prior injection of TILT-123, and all three patients achieved relatively long OS. Two out of three of these patients' biopsies were from non-injected tumors in the peritoneum. Together with the non-injected abdominal wall tumor from patient 20204, these observations suggest a dyslipidemia-associated, IgM-dependent mechanism that promotes the homing or persistence of TILT-123 immune complexes at these anatomical sites. One possible explanation is that natural IgM binding to adenovirus may redirect viral tropism toward Fc receptor-expressing cells, thereby circumventing factor X-mediated heparan sulfate-mediated liver targeting.[28] As an alternative mechanism, elevated secretion of natural IgM at these anatomical sites could facilitate viral trapping and sustained infection.

Given the likely formation of circulating immune complexes between IGVL8-61 IgM and TILT-123, modified LDL, or both, the antibody-to-antigen ratio is likely to be a critical factor achieving optimal therapeutic efficacy. This ratio likely influences the size and solubility of immune complexes, which in turn affects their clearance from circulation and their potential to accumulate in tissues and trigger immune responses. In terms of valency and immune signaling, it is well established that an excess of neither antigen nor antibody necessarily results in optimal Fc receptor engagement or effector function. If this hypothesis holds true, then in the context of TILT-123 therapy, it may be important to first consider the circulating concentration of IGLV8-61-encoded IgM, or other relevant Igs, when determining the optimal therapeutic dose of TILT-123. Alternatively, pre-forming immune complexes prior to administration, a strategy used in certain vaccine approaches, could be considered to improve systemic delivery or potentiate an immune response. This approach may be particularly efficacious against tumor types that actively internalize IgM-containing complexes, potentially via Fc receptor expression, or in metastatic sites characterized by elevated lipid metabolism, such as peritoneal tumors. It is conceivable that cancer cells within these specific microenvironments, driven by their metabolic demands, exploit IgM-oxLDL immune complexes as a metabolic resource, fueling proliferation and progression.

Natural IgM-producing B1 B cells and plasma cells predominantly reside within body cavities, notably the peritoneal and pleural spaces,

Figure 7. Dyslipidemia is associated with improved clinical outcomes across multiple phase 1 trials with TILT-123

(A) Treatment schedule for T115 cohort 7, T563-1a, and T215; created in BioRender (https://BioRender.com/kqkepx6). (B) Correlations between baseline serum concentrations of HNE-oxLDL with OS in T115 cohort 7, T563-1a, and T215. Patient 10107 from trial T215, who achieved a complete response, is highlighted.

(C–E) Kaplan-Meier curves illustrating OS stratified by baseline HNE-modified oxLDL concentrations (≥ median vs. < median) in patients from T115 (cohorts 1–7) (n = 26), T563-1a (n = 15), and T215 (n = 14). (F) Summary of overall response in all patients from T115 cohorts 1–7 and T563-1a, assessed according to both RECIST 1.1 and PET response criteria. (G) Summary of overall response in patients with a documented history of hyperlipidemia (as recorded in the eCRF) from cohorts T115-7, T563-1a, and T215, based on best response assessed by either RECIST 1.1 or PET criteria. (H) Kaplan-Meier curves illustrating OS in patients from T115 cohorts 1–7 and T563-1a, stratified by the presence or absence of a documented history of hyperlipidemia (n = 41). (I) Illustration of key clinical characteristics shared by the best-responding patients in T115, T215, and T563-1a, including the presence of dyslipidemia, number of prior chemotherapy lines, and anatomical location of the injected tumor (created in BioRender [https://BioRender.com/2qhthnt]). (J) Comparison of the maximum percentage reduction in CT-measured tumor size between tumors located in the abdomen or peritoneum and those in other anatomical locations in patients from T115 cohorts 1–7 and T563-1a. Statistical significance between groups was evaluated using a two-tailed Mann-Whitney U test. Differences in Kaplan-Meier survival curves were assessed using the log rank (Mantel-Cox) test. eCRF, electronic case report form. Statistical significance was defined as $p < 0.05$.

with a minor presence in the spleen and lymph nodes. This localization is heavily influenced by the peritoneal immune microenvironment, where resident peritoneal macrophages regulate B1 B cell migration and homing through chemokine expression.[30] Peritoneal macrophages are critical scavengers, recognized for their role in clearing pathogens and modified LDL, including the *in vitro* uptake of anionic modified LDL by murine peritoneal macrophages.[31] Notably, monocytes express scavenger receptors capable of mediating the uptake of both acetyl-LDL and adenovirus, suggesting a conserved clearance pathway.[32] In the context of dyslipidemia, B1 B cells and plasma cells generate natural IgM that binds to elevated levels of modified LDL, and these cells can recognize immune complexes via their Fc receptors. We hypothesize that this inherent mechanism, aimed at enhancing the clearance of modified LDL and associated complexes, may inadvertently facilitate the transport and accumulation of TILT-123 to the highly active immunological niches of the peritoneal and abdominal regions.

The precise role of IGLV8-61 IgM-TILT-123 immune complexes in modulating the function of IgM receptor-expressing immune cells, such as T cells, requires further mechanistic elucidation. A critical unknown is whether the observed phenomena reflect a passive distribution of these IgM-viral complexes or an active engagement mediated through immune complex-binding receptors on lymphocytes, which could trigger cellular redistribution or alter the antiviral activity of the T cells themselves. Significantly, clinical data from the TUNIMO trial suggest that a greater reduction in circulating lymphocyte counts post-treatment correlates with favorable clinical outcomes.[17] This observation supports the hypothesis of a treatment-induced lymphocyte redistribution, potentially facilitating the trafficking of TILT-123 to peripheral phagocytes and tumor sites. Preliminary analysis within our study lends further support: a greater fold decrease (day 8 vs. baseline) in circulating FcμR (FCMR)-expressing $CD8^+$ T cells is associated with longer OS. This finding is consistent with previous preclinical evidence suggesting that TILs can function as transient carriers for TILT-123.[33] The link between metabolic factors and these dynamics is also suggested by the finding that baseline levels of HNE-modified oxLDL (but not CML or MDA-oxLDL) showed a negative correlation with the lymphocyte count on day 8, potentially reinforcing the proposed immune-metabolic axis in viral delivery. Moreover, we observed a significant negative correlation between the abundance of IGLV8-61 and CD8a and other cytotoxic markers in this study, supporting an unexplored interplay involving IGVL8-61 IgM and CD8 T cells. Biodistribution studies using adoptive transfer of $FCMR^+$ lymphocytes and IGLV8-61 IgM-TILT-123 immune complexes would provide critical insights into their specific contribution to enhanced systemic delivery and therapeutic response. Additionally, further investigation into the roles of IGLV8-61 IgM and oxLDL in modulating the immune response to TILT-123 within the tumor microenvironment is warranted.

Among the modified LDL variants analyzed in this study, HNE-modified oxLDL consistently showed the strongest correlation with improved clinical outcomes. Although the precise mechanism underlying this association remains to be fully characterized, our current findings suggest favorable promotion of IGLV8-61 IgM secretion, plasma cell differentiation, and subsequent ADE of TILT-123. That is, oxLDL may serve as systemic immunogenic bait that induces the production of specific IgM clones (systemic imprinting). Consistent with this, we observed a strong significant positive correlation between the proportion of circulating IgM^+ plasma cells and OS in this study. Alternatively, oxLDL may favorably modulate the expression of relevant receptors-involved immune complex uptake. Another mechanism, and a possible contributor to improved efficacy, is competitive engagement of shared scavenger receptors involved in the clearance of both TILT-123 and modified LDL. Supporting this, HNE-modified proteins have been shown to inhibit the uptake of acetyl-LDL by the lectin-like oxLDL receptor-1 (LOX-1) in a dose-dependent manner.[34] Furthermore, studies in the context of adenovirus-mediated liver gene therapy suggest that lipoproteins may compete with adenoviruses for cellular entry, potentially through overlapping pathways involving the LDL receptor, lipoprotein receptor-related protein, and heparan sulfate proteoglycans.[35] Consistent with this hypothesis, we observed improved DC in patients exhibiting a higher percentage of macrovesicular steatosis in the liver. Since macrovesicular steatosis is a histological feature of steatotic liver disease (commonly associated with dyslipidemia and high circulating modified LDL), this finding suggests that the accumulation of lipids and modified lipoproteins in the liver may decrease TILT-123 hepatic sequestration by occupying key clearance receptors, thereby increasing the effective systemic dose available for tumor targeting. Multiple scavenger receptor families contribute to the recognition and clearance of LDL and its modified forms, with specificity influenced by oxidation state and surface charge and expression varying by cell type and anatomical location. Future studies integrating single-cell profiling of circulating immune cells together with liver and tumor biopsies, particularly from peritoneal and abdominal sites, will be essential to map cell-type-specific receptor expression and its impact on treatment efficacy. Additionally, investigating the effect of Ad5/3 chimerism compared to Ad5 or Ad3 alone on a particle's surface charge and its subsequent influence on scavenger receptor uptake and IGLV8-61 binding dynamics is warranted. Extending this analysis to other oncolytic viruses will also be important to determine whether similar IgM-binding interactions occur across platforms. One limitation of the present study was the inability to quantify other modified LDL species or perform direct binding assays with HNE-oxLDL due to the lack of commercially available reagents. Follow-up studies addressing these components will be critical to further elucidate their role in the mechanism of action.

Patient 10107 (complete response) from the T215 trial is a valuable example that highlights the importance of anti-modified LDL antibodies in the mechanism of action, suggesting that their role may be more critical than the presence of modified LDL. Using TCR prediction tools, we previously identified that the most expanded clone in the patient's TIL product, derived from a lymph node resection,

comprised T cell receptors predicted to be specific for apoB-100.[24] In this study, we confirmed their reactivity to modified LDL. Supporting this, a recent murine study demonstrated that adoptive transfer of $CD4^+$ T cells targeting apoB-100 promoted B cell activation and the production of anti-LDL Igs, which enhanced LDL clearance and reduced atherosclerotic lesions.[36] This may reflect a situation similar to what occurred in patient 10107, who had relatively low serum concentrations of all three variants of oxLDL. It is likely that patient 10107 has a high abundance of antibodies targeting LDL and its modified forms, leading to excessive clearance of circulating LDL particles. Notably, the patient had a history of intermittent epistaxis and von Willebrand disease. Given that oxLDL and OSEs are known to influence coagulation and that natural IgM can inhibit OSE-induced thrombosis, this observation may be mechanistically relevant.[15,16] In our clinical trials, prolonged coagulation time was associated with improved clinical outcomes. Furthermore, the abundance of IGLV8-61 correlated positively with aPTT, suggesting a possible link between anti-LDL immunity, coagulation dynamics, and clinical outcomes. Future studies should focus on quantifying serum antibodies that bind to individual modified LDL variants, to better understand their role in therapeutic response of OVs such as TILT-123.

In conclusion, our findings reveal a novel link between modified LDL, natural IgM, and therapeutic response to oncolytic viruses such as TILT-123. The association of HNE-modified LDL with clinical benefit highlights the potential of immunometabolic biomarkers to guide treatment stratification. Ongoing trials will be key to validating these insights and supporting biomarker-driven treatment strategies.

MATERIALS AND METHODS

Ethics statement

Clinical trials were performed by TILT Biotherapeutics Oy by permission of Finland/Fimea Klnro 49/2020, EC-HUS/1804/2020 (NCT04695327); Finland/Fimea permit Klnro 135/2021, USA/Food and Drug Administration permit Investigational New Drug number 27209, EC (Finland) TUKIJA/405/2021, Mayo Clinic institutional review board (USA) number 22-000078 (NCT05222932); EC (Denmark) 1905760, EC (France) CPPIDF1-2019-ND83 cat.1, (NCT04217473). Evaluation was conducted by ethics committees.

Informed consent was obtained from all patients reported in this trial. The study design and conduct complied with all relevant regulations regarding the use of human study participants and was conducted in accordance with the criteria set by the Declaration of Helsinki.

Patients

TUNIMO (NCT04695327) was a multi-center, single-arm, dose-escalation phase 1 trial designed to assess the safety, tolerability, efficacy, and immunogenicity of TILT-123 in advanced solid cancers (mixed tumor indications). Key inclusion criteria have been described previously.[1] A total of 20 patients were enrolled across six dosing cohorts in a 3 + 3 dose-escalation design. Patients received an initial single i.v. dose of TILT-123 (ranging from 3×10^9 to 4×10^{12} virus particles) followed by up to five doses of i.t. TILT-123 (ranging from 3×10^9 to 5×10^{11} virus particles). Patients were assessed with imaging at baseline and on day 78 with contrast-enhanced CT and fluorodeoxyglucose PET using RECIST 1.1, iRECIST, and PET-based response criteria.

Sample collection

Serum was collected using CAT Vacutainer (Becton Dickinson, Franklin Lakes, NJ, catalog no. 369032) on multiple days during the trial, including pre-treatment (0 h) and 16 h post-treatment samples on days 1, 8, 22, 36, 50, and 64. Samples collected on day 1 (0 h), day 1 (16 h), and day 8 (0 h) were the focus of the present study. All samples were frozen at −20°C immediately upon collection and transferred to −20°C within 4 weeks for storage. Standard laboratory blood tests for CBC, liver and kidney function tests, and standard electrolytes were evaluated using standard hospital laboratory equipment.

Trypsin digestion

Serum samples were desalted using Zeba Spin desalting plates (Thermo Fisher Scientific, Waltham, MA) prior to 50-μL aliquots being depleted of excess albumin using the Pierce albumin depletion kit (Thermo Fisher Scientific). The remaining protein concentration was determined using the Bradford MX Protein Assay (Expedeon, Heidelberg, Germany), after which the samples were dried and resuspended with 35 μL 6 M urea. Then, as previously described by Kontro et al., reduced and alkylated prior to trypsin digestion.[37] The samples were diluted 1:10 with a 50-mM Tris solution to reduce the urea concentration; trypsin (from bovine pancreas [Sigma-Aldrich, St. Louis, MO]) was added at a mass ratio of 1:50 and incubated at 37°C overnight.

Size-exclusion chromatography

Size-exclusion chromatography was performed as previously described.[7] Dried trypsinized samples were resuspended in 55 μL 0.1% formic acid (FA) (LiChropur FA 98%–100% for high-performance liquid chromatography [Merck, Darmstadt, Germany]), centrifuged to pellet any precipitated molecules. Then, 50 μL of each supernatant was loaded into the size-exclusion column (GE Healthcare Superdex peptide 3.2/300), and was run at an isocratic gradient of 0.05 mL/min of 0.1% FA. To determine the correct fractions, we originally collected fractions at 1-min intervals from a pooled serum sample from 17 to 42 min, after which we screened each 1-min fraction using ultra-performance liquid chromatography-elevated energy MS (UPLC-MS^E). We detected glycopeptides from 20 to 32 min and peptides from 30 to 39 min. We then performed size-exclusion chromatography on 50 μL of each digest, collecting from each serum sample two aliquots: one containing glycopeptide and one containing peptides, which then were dried. We depleted the top 12 peptides to allow inclusion of the Igs.

MS

The nanoAcquity UPLC system (Waters Corporation, Milford, MA), joined to the Synapt G2-Si HDMS (Waters Corporation), was used, and the TRIZAIC nanotile (Waters Corporation) was used as a separating device prior to MS of both N-glycopeptide- and peptide-enriched fractions. Calibration was performed with sodium iodide clusters over a mass range of 50–2,500*m/z*. The whole study was done in the positive mode. Leucine enkephalin ($C_{25}H_{37}O_7$, M+H$^+$, 556.2771 *m/z*, 1 ng/μL in 50:50 acetonitrile:water + 0.1% FA) was infused into the reference sprayer at 300 nL/min to act as a lock mass for MS data analysis.

Samples were kept at −20°C until they were placed in the sample manager at 4°C for up to 24 h. UPLC was used for all sample acquisitions. For LC, we used buffer A (0.1% FA in UPLC-grade water) and buffer B (0.1% FA in ACN). UPLC analytical gradients are shown in Table S1.

Proteomics

Ultra definition elevated collision energy mass spectrometry (UDMS^E) in resolution mode was used for protein quantitation and identification.

MS data analysis

Both peptide quantification/identification and glycopeptide quantification raw files were imported to Progenesis QI for proteomics (Nonlinear Dynamics, Newcastle upon Tyne, UK). Post-acquisition mass correction was done when the raw data were imported to Progenesis with lock mass ion of M + H+ 556.2771 *m/z*.

For the proteomics project, peptides were aligned and normalized with Hi3 *Escherichia coli* peptides (Waters Corporation).[38] For peptide identification and quantification, ion accounting was used, against the UniProt human database, search parameters on the Protein Lynx Global Server (PLGS) version 3.03 search engine are as follows: cleaved by trypsin; two missed cleavages allowed; maximum protein was 250 kDa; fixed modification: carbamidomethyl cystine; variable modification: oxidation methionine; automatic tolerance parameters for peptide and fragment tolerance; maximal false discovery rate (FDR) 1%; and default parameters for ion-matching requirements. Quantification using non-conflicting peptides was applied. The parsimony principle was used for protein grouping in the case of shared peptides. Only proteins with two or more unique peptides were included.

Supporting correlative data

The data on anti-adenovirus nAbs, TILT-123 DNA in blood, and Olink serum proteomics were previously published.[1,17] In brief, nAbs were quantified using a previously described cell-based luciferase assay. Detectable nAb titers were defined as those greater than a 1:64 dilution. The nAb assay specifically measures antibody-mediated neutralizing activity as complement is inactivated during sample processing by heat treatment. Serum proteomic analysis was carried out using the Olink Immuno-Oncology assay (Thermo Fisher). The readout is expressed as NPX, Olink's proprietary unit for quantifying relative protein abundance. NPX values are generated using Olink's Proximity Extension Assay platform, which converts raw qPCR or next-generation sequencing signal data into a consistent and comparable unit.

Quantification of oxLDL in serum

Serum oxLDLs were quantified from patients in the TUNIMO, PROTA, and TUNINTIL trials using the following commercially available kits according to the manufacturer's instructions:

Oxidized LDL Assay Kit, HNE-LDL, Human (Abcam, Cambridge, UK; ab242304)

Oxidized LDL Assay Kit, MDA-LDL, Human (Abcam; ab242302)

Oxidized LDL Assay Kit, CML-LDL, Human (Abcam; ab242303)

Flow cytometry of PBMCs

Cryopreserved PBMCs were thawed, washed with 10% fetal bovine serum RPMI, and seeded at a density of 1×10^6 cells per well. After resting for 1 h in a 37°C incubator, the cells were stained with antibodies targeting CD3, CD8, CD19, CD14, CD27, CD38, CD43, CD5, CD204, IgM, IgD, and FCMR for 45 min at 4°C. More details on the antibodies can be found in Table S5. Fluorochrome compensation was performed using UltraComp eBeads Compensation Beads (Thermo Fisher, catalog no. 01-2222-42). Samples were acquired using Novocyte Quanteon (Agilent Technologies, Santa Clara, CA), and generated fcs-files were analyzed with FlowJo (BD Life Sciences, Franklin Lakes, NJ).

Single-cell BCR sequencing

The data were published previously by our group.[23] Single-cell capture and library preparation were performed using the BD Rhapsody Single-Cell RNA Sequencing pipeline (BD Biosciences, Franklin Lakes, NJ). PBMCs were captured following labeling with the Single-Cell Multiplexing Kit (BD Biosciences, catalog no. 633781). Library preparation was performed using the BD Rhapsody Human Immune Response Targeted amplification kit (BD Biosciences, catalog no. 633750) and the BD Rhapsody TCR/BCR Full-Length Amplification kit (BD Biosciences, catalog no. 665345) according to the manufacturer's instructions. TCR/BCR libraries were sequenced using the NovaSeq 6000 platform (Illumina, San Diego, CA) to generate 250-bp paired-end reads, while mRNA and sample tag libraries were sequenced using the NovaSeq X Plus platform (Illumina) to generate 150-bp paired-end reads. FASTQ files were processed using the BD Rhapsody Sequence Analysis Pipeline run (pipeline run via the Seven Bridges Genomics cloud platform) and loaded into the R package Seurat (version 5.0.0) for further analysis.

Mammalian expression of IgM

Full-length BCR sequences from three IGLV8-61 clones (CPK1031, CPK1032, and CPK1033) were reconstructed, and recombinant pentameric IgMs were synthesized using the TurboCHO Antibody Expression platform (GenScript, Piscataway, NJ). IgMs were purified

using POROS CaptureSelect IgM Affinity Matrix and stored in PBS, pH 7.2, until further use (GenScript).

IgM binding studies

Indirect ELISAs were developed to evaluate the binding of CPK1031, CPK1032, and CPK1033 to TILT-123, LDL (Thermo Fisher, catalog no. L3486), acetyl-LDL (Thermo Fisher, catalog no. L35354), CML-oxLDL (Thermo Fisher, catalog no. L34357), and MDA-oxLDL (Cell Biolabs, San Diego, CA, catalog no. STA-212). Immuno Standard Black PolySorp plates (Thermo Fisher, catalog no. 475523) were coated with either 5 μg/mL LDL/acetyl-LDL/oxLDL or 1E+8 VP/well of TILT-123 in carbonate-bicarbonate buffer (Sigma-Aldrich, catalog no. C3041) overnight. The next day, wells were blocked with 3% fatty acid-free BSA (Sigma-Aldrich, catalog no. A8806) for 1–2 h at room temperature. Wells were washed with PBS and then incubated with either CPK1031, CPK1032, or CPK1033 following a 2-fold step dilution for 1 h in 1% fatty-acid free BSA. Wells were washed three times and then incubated with goat anti-human IgM (heavy chain) cross-adsorbed secondary antibody and Alexa Fluor 488 (Thermo Fisher, catalog no. A-21215) for 1 h in 1% fatty acid-free BSA. After washing, the plates were read using the Hidex Sense plate reader (Hidex, Turku, Finland).

Transduction assay

A549, SCOV3, and SW620 cells were seeded at 10,000 cells per well in 96-well plates. On the following day, 10, 100, or 1,000 VP/cell of non-replicative Ad5/3-Luc was pre-complexed with 0, 3.125, 6.25, 12.5, 25, or 50 μg/mL CPK1031, CPK1032, or CPK1033 prior to cell treatment. The following day, luciferase activity was quantified by luminescence using the Promega Luciferase Assay System (Madison, WI), following the same protocol as our previously reported nAb assay.[39]

Cell viability assay

A549, SCOV3, and SW620 cells were seeded at 10,000 cells per well in 96-well plates. On the following day, 10, 100, or 1,000 VP/cell of Ad5/3-d24-E2F (TILT-123 unarmed backbone virus) was pre-complexed with 0, 3.125, 6.25, 12.5, 25, or 50 μg/mL CPK1031, CPK1032, or CPK1033 prior to cell treatment. Cell viability was assessed on day 3 using the CellTiter 96 AQueous One Solution Proliferation Assay (Promega) at 20% reagent volume, following the manufacturer's instructions. Absorbance was measured at 490 nm using a Hidex Sense reader, and data were corrected for background.

Blocking studies

A549 cells were seeded at 10,000 cells per well in 96-well plates. On the following day, 5 μg/mL of either of the following blocking antibodies or respective isotype controls were added to cells in a volume of 100 μL per well: anti-CD36 (STEMCELL Technologies, Vancouver, Canada, catalog no. 50-197-5477), anti-LOX1/ORL1/golocdacimab (MedChemExpress, Monmouth Junction, NJ, catalog no. HY-P99646), anti-CD204/SRAI (Bio-Techne, Minneapolis, MN, catalog no. NBP1-00092), anti-CD206/MMR (Invitrogen, Waltham, MA, catalog no. MA5-28581), anti-FcμR/FAIM3 (BioLegend, San Diego, CA, catalog no. 398102), anti-Fcα/μR/CD351 (BioLegend, catalog no. 137308), anti-pIgR (Thermo Fisher, catalog no. PA5-35340), anti-pIgR (Abcam, catalog no. ab96196), rabbit IgG isotype control (Invitrogen, catalog no. 10500C), mouse IgG2b isotype control (Invitrogen, catalog no. 02-6300), mouse IgG1 isotype control (Invitrogen, catalog no. 02-6100), and human IgG1κ from human myeloma plasma (Merck, catalog no. I5154-1MG). After a 20-min incubation, 100 VP/cell of Ad5/3-Luc, pre-complexed with 0, 3.125, 6.25, 12.5, 25, or 50 μg/mL CPK1033 was added, resulting in a final blocking antibody concentration of 2.5 μg/mL. The following day, luciferase activity was quantified by measuring luminescence, as described in the transduction assay.

IHC

The anti-pIgR antibody (Abcam, catalog no. ab96196) was used at a 1:500 dilution to detect pIgR expression in A549 spheroids. To generate spheroids, A549 cells were seeded at 10,000 cells per well in Nunclon Sphera 96-well, Sphera-treated, U-shaped bottom microplates (Thermo Fisher, catalog no. 174929). After 7 days, the spheroids were pooled prior to cytoblock preparation, sectioning, and staining.

Human IFN-γ ELISpot

To assess the reactivity of TILs against native LDL and modified forms of LDL, 10,000 PBMCs together with 5,000 TIL infusion products were co-cultured with either LDL, acetyl-LDL, CML-oxLDL, MDA-oxLDL, or alone using the human IFN-γ ELISpot (Bio-Techne, catalog no. EL285). The following day, the plates were processed according to the manufacturer's instructions and sent for scanning and analysis (CTL Europe GmbH, Rutesheim, Germany).

Validation patient population

Patients treated in PROTA (NCT05271318) and TUNINTIL (NCT04217473) were included to validate findings from the analysis of TUNIMO and have previously been reported.[1–3]

Statistical analysis

The initial statistical analysis of groups was calculated using one-way ANOVA and t test assuming normally distributed data. Multiple testing (FDR) was performed using the Benjamini-Hochberg method, with the FDR set to <0.05.

The normality of the data was assessed using the Shapiro-Wilk test. Unpaired t test was used for group comparison when data were normally distributed, while the non-parametric Mann-Whitney test was used to compare non-normally distributed data. Two-way ANOVA was used to perform mixed-effects analysis in binding studies. Correlations between variables were calculated using either Pearson correlation or Spearman's rank correlation coefficient. Survival analysis was calculated using the log rank (Mantel-Cox) test. Enrichment analysis was performed using Reactome.[40] Data were visualized in GraphPad Prism version 10.4.2 (GraphPad Software, La Jolla, CA).

Graphical illustrations

Illustrations in Figures 1A, 5B–5D, 6A, and 6I, in addition to the graphical abstract, were created in BioRender (https://BioRender.

com). Publication licenses can be found in the figure legends of the respective figure.

DATA AND CODE AVAILABILITY

Source data are provided with this paper in Table S1. The MS proteomics data have been deposited to the ProteomeXchange Consortium via the PRIDE partner repository with the dataset identifier PXD054594 (ProteomeXchange Consortium: PXD054594) and 10.6019/PXD054594.[41]

ACKNOWLEDGMENTS

The authors thank the patients enrolled in the study and their families. Olink biomarker analysis was performed by the FIMM Genomics Targeted Biomarker Assays unit at the University of Helsinki supported by HiLIFE and Biocenter Finland. We are grateful for the valuable contributions of the FIMM Digital Microscopy and Molecular Pathology Unit supported by HiLIFE and Biocenter Finland for IHC services. We thank Minna Oksanen, Sini Raatikainen, and Tobias Loftin for their expert assistance. This study was supported by the Jane and Aatos Erkko Foundation, EU Horizon Grant 811693 (UNLEASHAD) (to A.H.), the Finnish Cultural Foundation (to J.H.A.C., D.C.A.Q., E.J., and S.B.), the EU Horizon 2020 Research and Innovation Programme under the Marie Skłodowska-Curie Grant Agreements (no. 813453) (to A.H. and J.H.A.C.), TILT Biotherapeutics, HUCH Research Funds (VTR) (to A.H., O.H., and A.K.), Cancer Foundation Finland (to A.H., J.H.A.C., and T.V.K.), the Sigrid Juselius Foundation (to A.H.), the K. Albin Johansson Foundation (to J.H.A.C. and S.B.), Selma and Maja-Lisa Selander's Fund for Research in Odontology (Minerva Foundation) (to J.H.A.C.), the Ida Montinin Foundation (to J.H.A.C., T.V.K., and S.B.), and the Finnish Red Cross Blood Service (to A.H.). We thank Albert Ehrnrooth and Karl Fazer for research support. We thank the Department of Defense for supporting this study (OC220391) (to A.H.). TILT Biotherapeutics Oy funded the study design, data collection, and analysis and was involved in the study design, data analysis and interpretation, writing, and submission of the report for publication. The study ethics were reviewed by the Helsinki and Uusimaa Hospital District Ethical Board (statement HUS/1804/2020). The clinical trial protocol was reviewed by the Finnish Medical Agency (approval 49/2020). All patients enrolled in the study gave informed consent.

AUTHOR CONTRIBUTIONS

J.H.A.C., S.A.P., S.J., T.V.K., T.T., V.A., D.C.A.Q., A.P., and N.S., conceptualization, design, investigation, methodology, acquisition, data curation, analysis, interpretation, visualization, drafting, and editing; E.J., M.v.d.H., L.H., N.O., S.B., A.E., J.D.F., and S.H.; acquisition, data curation, analysis, and editing; S.Z., S.G.-V.-K., A.K., and O.H., administrative, technical, or material support; I.M.S., J.M., K.J., M.S.B., T.A., T.M., C.K., R.H., S.S., J.M.d.S., V.C.-C., R.R., and A.H., conceptualization, design, investigation, methodology, acquisition, interpretation, drafting, and editing.

DECLARATION OF INTERESTS

A.H. is a shareholder in Circio Holdings ASA. A.H., A.K., C.K., J.H.A.C., J.M.d.S., D.C.A.Q., S.S., R.H., L.H., and V.C.-C. are employees and shareholders in TILT Biotherapeutics Oy. M.S.B. is on the TILT Biotherapeutics scientific advisory board. K.J. has held advisory roles in Bayer AG, Bristol-Myers Squibb, MSD, Roche, and Ipsen and is a shareholder in Faron Pharmaceuticals.

SUPPLEMENTAL INFORMATION

Supplemental information can be found online at https://doi.org/10.1016/j.ymthe.2026.01.019.

REFERENCES

1. Pakola, S.A., Peltola, K.J., Clubb, J.H.A., Jirovec, E., Haybout, L., Kudling, T.V., Alanko, T., Korpisaari, R., Juteau, S., Jaakkola, M., et al. (2024). Safety, efficacy, and biological data of T-cell–enabling oncolytic adenovirus TILT-123 in advanced solid cancers from the TUNIMO monotherapy phase I trial. Clin. Cancer Res. *30*, 3715–3725.
2. Block, M.S., Clubb, J.H.A., Mäenpää, J., Pakola, S., Quixabeira, D.C.A., Kudling, T., Jirovec, E., Haybout, L., van der Heijden, M., Zahraoui, S., et al. (2025). The oncolytic adenovirus TILT-123 with pembrolizumab in platinum resistant or refractory ovarian cancer: the phase 1a PROTA trial. Nat. Commun. *16*, 1381.
3. Monberg, T.J., Pakola, S.A., Albieri, B., Ellebaek, E., Donia, M., Eefsen, R.L., Borch, T.H., Kudling, T.V., Lorentzen, T., Hendel, H.W., et al. (2025). Safety and efficacy of combined treatment with tumor-infiltrating lymphocytes and oncolytic adenovirus TILT-123 in metastatic melanoma. Cell Rep. Med. *6*, 102016.
4. Jirovec, E., Quixabeira, D.C.A., Clubb, J.H.A., Pakola, S.A., Kudling, T., Arias, V., Haybout, L., Jalkanen, K., Alanko, T., Monberg, T., et al. (2024). Single intravenous administration of oncolytic adenovirus TILT-123 results in systemic tumor transduction and immune response in patients with advanced solid tumors. J. Exp. Clin. Cancer Res. *43*, 297.
5. Ling, A.L., Solomon, I.H., Landivar, A.M., Nakashima, H., Woods, J.K., Santos, A., Masud, N., Fell, G., Mo, X., Yilmaz, A.S., et al. (2023). Clinical trial links oncolytic immunoactivation to survival in glioblastoma. Nature *623*, 157–166.
6. Qiu, Q., Xu, Z., Tian, J., Moitra, R., Gunti, S., Notkins, A.L., and Byrnes, A.P. (2015). Impact of natural IgM concentration on gene therapy with adenovirus type 5 vectors. J. Virol. *89*, 3412–3416.
7. Dickinson, A., Saraswat, M., Joenväärä, S., Agarwal, R., Jyllikoski, D., Wilkman, T., Mäkitie, A., and Silén, S. (2020). Mass spectrometry–based lipidomics of oral squamous cell carcinoma tissue reveals aberrant cholesterol and glycerophospholipid metabolism—A Pilot study. Transl. Oncol. *13*, 100807.
8. Koski, A., Ahtinen, H., Liljenback, H., Roivainen, A., Koskela, A., Oksanen, M., Partanen, K., Laasonen, L., Kairemo, K., Joensuu, T., et al. (2013). (18) F]-fluorodeoxyglucose positron emission tomography and computed tomography in response evaluation of oncolytic adenovirus treatments of patients with advanced cancer. Hum. Gene Ther. *24*, 1029–1041.
9. Pappas, L., Foglierini, M., Piccoli, L., Kallewaard, N.L., Turrini, F., Silacci, C., Fernandez-Rodriguez, B., Agatic, G., Giacchetto-Sasselli, I., Pellicciotta, G., et al. (2014). Rapid development of broadly influenza neutralizing antibodies through redundant mutations. Nature *516*, 418–422.
10. Schultheiß, C., Willscher, E., Paschold, L., Ackermann, C., Escher, M., Scholz, R., Knapp, M., Lützkendorf, J., Müller, L.P., Schulze Zur Wiesch, J., et al. (2024). B cells expressing mutated IGHV1-69–encoded antigen receptors related to virus neutralization show lymphoma-like transcriptomes in patients with chronic HCV infection. Hepatol. Commun. *8*, e0503.
11. He, P., Liu, B., Gao, X., Yan, Q., Pei, R., Sun, J., Chen, Q., Hou, R., Li, Z., Zhang, Y., et al. (2022). SARS-CoV-2 Delta and Omicron variants evade population antibody response by mutations in a single spike epitope. Nat. Microbiol. *7*, 1635–1649.
12. Johansen, F.E., Braathen, R., and Brandtzaeg, P. (2000). Role of J chain in secretory immunoglobulin formation. Scand. J. Immunol. *52*, 240–248.
13. Lydyard, P.M., Youinou, P.Y., and Cooke, A. (1987). CD5-positive B cells in rheumatoid arthritis and chronic lymphocytic leukemia. Immunol. Today *8*, 37–39.
14. Cronstein, B.N., and Aune, T.M. (2020). Methotrexate and its mechanisms of action in inflammatory arthritis. Nat. Rev. Rheumatol. *16*, 145–154.
15. Obermayer, G., Afonyushkin, T., Göderle, L., Puhm, F., Schrottmaier, W., Taqi, S., Schwameis, M., Ay, C., Pabinger, I., Jilma, B., et al. (2021). Natural IgM antibodies inhibit microvesicle-driven coagulation and thrombosis. Blood J. Am. Soc. Hematol. *137*, 1406–1415.
16. Obermayer, G., Afonyushkin, T., Puhm, F., Schwameis, M., Jilma, B., and Binder, C.J. (2016). Natural IgM Against Oxidation-Specific Epitopes Inhibit Microvesicle-Driven Coagulation. Blood *128*, 2562.
17. Pakola, S.A., Clubb, J.H.A., Kudling, T.V., van der Heijden, M., Jirovec, E., Arias, V., Haybout, L., Peltola, K., Alanko, T., Sormunen, J., et al. (2025). Transient lymphocyte count decrease correlates with oncolytic adenovirus efficacy in humans: mechanistic and biomarker findings from TUNIMO phase I trial. J. Immunother. Cancer *13*, e010493.
18. Taipale, K., Tähtinen, S., Havunen, R., Koski, A., Liikanen, I., Pakarinen, P., Koivisto-Korander, R., Kankainen, M., Joensuu, T., Kanerva, A., et al. (2018). Interleukin 8 activity influences the efficacy of adenoviral oncolytic immunotherapy in cancer patients. Oncotarget *9*, 6320–6335.
19. Terkeltaub, R., Banka, C.L., Solan, J., Santoro, D., Brand, K., and Curtiss, L.K. (1994). Oxidized LDL induces monocytic cell expression of interleukin-8, a chemokine with T-lymphocyte chemotactic activity. Arteriosclerosis and Thrombosis. Arterioscler. Thromb. *14*, 47–53.

20. Binder, C.J., Hartvigsen, K., Chang, M.K., Miller, M., Broide, D., Palinski, W., Curtiss, L.K., Corr, M., and Witztum, J.L. (2004). IL-5 links adaptive and natural immunity specific for epitopes of oxidized LDL and protects from atherosclerosis. J. Clin. Invest. *114*, 427–437.

21. Poss, K.D., and Tonegawa, S. (1997). Reduced stress defense in heme oxygenase 1-deficient cells. Proc. Natl. Acad. Sci. USA *94*, 10925–10930.

22. Ishikawa, K., Navab, M., Leitinger, N., Fogelman, A.M., and Lusis, A.J. (1997). Induction of heme oxygenase-1 inhibits the monocyte transmigration induced by mildly oxidized LDL. J. Clin. Invest. *100*, 1209–1216.

23. Kudling, T.V., Bychkov, D., Clubb, J.H.A., Pakola, S.A., Arias, V., Jirovec, E., van der Heijden, M., Ojala, N., Quixabeira, D.C.A., Haybout, L., et al. (2025). Single-cell profiling of peripheral blood mononuclear cells from patients treated with oncolytic adenovirus TILT-123 reveals baseline immune status as a predictor of therapy outcomes. Cancer Gene Ther. *32*, 649–661.

24. Monberg, T.J., Kudling, T., Albieri, B., Pakola, S., Ellebaek, E., Donia, M., Eefsen, R.L., von Buchwald, C., Kistler, C., Santos, J.M., et al. (2024). Durable complete response after combined treatment with tumor-infiltrating lymphocytes and oncolytic adenovirus (TILT-123) in a patient with metastatic mucosal melanoma. Immunooncol. Technol. *24*, 100726.

25. Taipale, K., Liikanen, I., Koski, A., Heiskanen, R., Kanerva, A., Hemminki, O., Oksanen, M., Grönberg-Vähä-Koskela, S., Hemminki, K., Joensuu, T., et al. (2016). Predictive and prognostic clinical variables in cancer patients treated with adenoviral oncolytic immunotherapy. Mol. Ther. *24*, 1323–1332.

26. Graf, R., Seagal, J., Otipoby, K.L., Lam, K.P., Ayoub, S., Zhang, B., Sander, S., Chu, V.T., and Rajewsky, K. (2019). BCR-dependent lineage plasticity in mature B cells. Science *363*, 748–753.

27. Xu, Z., Tian, J., Smith, J.S., and Byrnes, A.P. (2008). Clearance of adenovirus by Kupffer cells is mediated by scavenger receptors, natural antibodies, and complement. J. Virol. *82*, 11705–11713.

28. Xu, Z., Qiu, Q., Tian, J., Smith, J.S., Conenello, G.M., Morita, T., and Byrnes, A.P. (2013). Coagulation factor X shields adenovirus type 5 from attack by natural antibodies and complement. Nat. Med. *19*, 452–457.

29. Allen, R.J., and Byrnes, A.P. (2019). Interaction of adenovirus with antibodies, complement, and coagulation factors. FEBS Lett. *593*, 3449–3460.

30. Ansel, K.M., Harris, R.B.S., and Cyster, J.G. (2002). CXCL13 is required for B1 cell homing, natural antibody production, and body cavity immunity. Immunity *16*, 67–76.

31. Goldstein, J.L., Ho, Y.K., Basu, S.K., and Brown, M.S. (1979). Binding site on macrophages that mediates uptake and degradation of acetylated low density lipoprotein, producing massive cholesterol deposition. Proc. Natl. Acad. Sci. USA *76*, 333–337.

32. Li, P., Feng, F., Pan, E., Fan, X., Yang, Q., Guan, M., Chen, L., and Sun, C. (2018). Scavenger receptor-mediated Ad5 entry and acLDL accumulation in monocytes/macrophages synergistically trigger innate responses against viral infection. Virology *519*, 86–98.

33. Santos, J., Heiniö, C., Quixabeira, D., Zafar, S., Clubb, J., Pakola, S., Cervera-Carrascon, V., Havunen, R., Kanerva, A., and Hemminki, A. (2021). Systemic delivery of oncolytic adenovirus to tumors using tumor-infiltrating lymphocytes as carriers. Cells *10*, 978.

34. Kumano-Kuramochi, M., Shimozu, Y., Wakita, C., Ohnishi-Kameyama, M., Shibata, T., Matsunaga, S., Takano-Ishikawa, Y., Watanabe, J., Goto, M., Xie, Q., et al. (2012). Identification of 4-hydroxy-2-nonenal–histidine adducts that serve as ligands for human lectin-like oxidized LDL receptor-1. Biochem. J. *442*, 171–180.

35. Kivelä, A.M., Huusko, J., Gurzeler, E., Laine, A., Dijkstra, M.H., Dragneva, G., Andersen, C.B.F., Moestrup, S.K., and Ylä-Herttuala, S. (2017). High plasma lipid levels reduce efficacy of adenovirus-mediated gene therapy. Sci. Rep. *7*, 386.

36. Gisterå, A., Klement, M.L., Polyzos, K.A., Mailer, R.K.W., Duhlin, A., Karlsson, M.C.I., Ketelhuth, D.F.J., and Hansson, G.K. (2018). Low-density lipoprotein-reactive T cells regulate plasma cholesterol levels and development of atherosclerosis in humanized hypercholesterolemic mice. Circulation *138*, 2513–2526.

37. Kontro, H., Joenväärä, S., Haglund, C., and Renkonen, R. (2014). Comparison of sialylated N-glycopeptide levels in serum of pancreatic cancer patients, acute pancreatitis patients, and healthy controls. Proteomics *14*, 1713–1723.

38. Silva, J.C., Gorenstein, M.V., Li, G.Z., Vissers, J.P.C., and Geromanos, S.J. (2006). Absolute quantification of proteins by LCMSE: A virtue of parallel MS acquisition* S. Mol. Cell. Proteomics *5*, 144–156.

39. Hemminki, A., Wang, M., Desmond, R.A., Strong, T.V., Alvarez, R.D., and Curiel, D.T. (2002). Serum and ascites neutralizing antibodies in ovarian cancer patients treated with intraperitoneal adenoviral gene therapy. Hum. Gene Ther. *13*, 1505–1514.

40. Milacic, M., Beavers, D., Conley, P., Gong, C., Gillespie, M., Griss, J., Haw, R., Jassal, B., Matthews, L., May, B., et al. (2024). The Reactome Pathway Knowledgebase 2024. Nucleic Acids Res. *52*, D672–D678. https://doi.org/10.1093/nar/gkad1025.

41. Perez-Riverol, Y., Bai, J., Bandla, C., García-Seisdedos, D., Hewapathirana, S., Kamatchinathan, S., Kundu, D.J., Prakash, A., Frericks-Zipper, A., Eisenacher, M., et al. (2022). The PRIDE database resources in 2022: a hub for mass spectrometry-based proteomics evidences. Nucleic Acids Res. *50*, D543–D552.

YMTHE, Volume *34*

Supplemental Information

Dyslipidemia-associated natural IgM improves oncolytic virus TILT-123 efficacy through antibody-dependent enhancement in solid tumors

James Hugo Armstrong Clubb, Santeri Artturi Pakola, Sakari Joenväärä, Tatiana Viktorovna Kudling, Tiialotta Tohmola, Victor Arias, Elise Jirovec, Mirte van der Heijden, Dafne Carolina Alves Quixabeira, Annukka Pasanen, Lyna Haybout, Nea Ojala, Saru Basnet, Arianna Eleuteri, Julia Davila Ferrero, Stella Hirvenoja, Inge Marie Svane, Johanna Mäenpää, Katriina Jalkanen, Matthew Stephen Block, Tuomo Alanko, Tine Monberg, Sanae Zahraoui, Susanna Grönberg-Vähä-Koskela, Natasha Salmelin, Claudia Kistler, Riikka Havunen, Suvi Sorsa, João Manuel dos Santos, Victor Cervera-Carrascon, Anna Kanerva, Otto Hemminki, Risto Renkonen, and Akseli Hemminki

Table S1. Data file containing source data used in this study. See separate excel file.

Table S2. Patient characteristics and responses. F = female, M = male. NSCLC = non-small cell lung cancer, HSGOC = High Grade Serous Ovarian Cancer, NA = not available, PD = progressive disease, SD = stable disease, PR = partial response, PMD = progressive metabolic disease, SMD = stable metabolic disease, PMR = partial metabolic response, * = patient alive at data-cutoff.

Patient ID	Cohort	Sex	ECOG	Tumour Type	Stage	No. prior systemic treatments	RECIST 1.1	PET Respons e	Overall Survival (Days)
20202	1	F	1	NSCLC	IV	5	PD	PMR	102
20203	1	F	1	Melanoma of unknown primary	IV	7	NA	NA	66
20204	2	M	0	Myxoid liposarcoma	IV	5	PD	SMD	821
20205	2	F	1	Ovarian Serous Cystadenocarcinoma	IV	14	NA	NA	70
20101	2	F	1	Breast carcinoma	IV	7	NA	NA	56
20206	2	F	1	HGSOC	IIIC	16	PD	SMD	192
20103	3	M	0	Anaplastic Thryoid carcinoma	IV	1	PR	PMR	814*
20102	3	M	1	Rhabdomyosarcoma	IV	3	NA	NA	118
20211	3	F	1	Cutaneous melanoma	IV	3	PD	PMD	377
20212	4	F	1	Leiomyosarcoma	IV	4	PD	PMD	557
20104	4	M	1	Leiomyosarcoma	IV	6	PD	SMD	206
20107	4	M	0	Chondrosarcoma	IV	2	NA	NA	104
20213	5	M	1	Neuroendocrine carcinoma of the bladder	IV	3	NA	NA	94
20108	5	F	1	Adenoid cystic carcinoma	IV	0	SD	SMD	221
20214	5	F	1	Mucinous carcinoma of the appendix	IV	6	NA	NA	109
20216	6	F	1	Cutaneous melanoma	IV	2	NA	NA	104
20109	6	F	0	Leiomyosarcoma	IV	5	NA	NA	109
20217	6	F	1	Myxoid liposarcoma	IV	4	PD	PMD	131
20219	6	M	1	High grade mucoepidermoid carcinoma of the parotid gland	IV	2	PD	PMD	239
20111	6	F	0	High grade serous carcinoma of the peritoneum	IV	15	NA	NA	196

Table S3. PET criteria.

Complete metabolic response (CMR)	Complete resolution of FDG activity within measurable lesions and all reliably assessable lesions to background levels. No new FDGavid lesions in pattern typical of cancer*. Lymph nodes may remain metabolically active due to immune response (activated lymphocytes take up FDG).
Partial metabolic response (PMR)	> 30 % reduction in FDG activity measured as the summed SUVmax of measurable lesions (up to five lesions, max 2/organ). No new FDGavid lesions in pattern typical of cancer*.
Minor metabolic response (MMR)	10-29 % reduction in FDG summed SUVmax. No new FDG-avid lesions in pattern typical of cancer*.
Stable metabolic disease (SMD)	0-9 % reduction or up to < 30 % increase in FDG summed SUVmax. No new FDG-avid lesions in pattern typical of cancer*.
Progressive metabolic disease (PMD)	≥ 30 % increase in FDG summed SUVmax in pattern typical of tumor, or new clearly FDG-avid clinically significant lesions in pattern typical of cancer*. Increase in metabolic activity in lymph nodes should not result in PMD if no progression is detected elsewhere, since it might reflect immunological activation and not progression.
*"clinically significant lesions in pattern typical of cancer" are defined as lesions associated with a CT abnormality most consistent with cancer (≥ 2 cm in diameter), and clearly not because of inflammation of infection or related to treatment response.	

Table S4. IGLV8-61 containing clones detected from full length BCR sequencing. Including patient ID, day of sample collection, heavy chain variable (VH) gene, joining heavy (JH) gene, diversity heavy chain (DH), heavy chain CDR3 sequence, variable light (VL) gene, joining light (JL) gene, light chain CDR3 sequence and Chain (isotype). NA indicates where information was not available. IGLV8-61*01 is highlighted in the green column. BL = baseline.

#	Patient ID	Day	VH gene	DH gene	JH gene	Heavy chain CDR3	VL gene	JL gene	Light chain CDR3	Constant region
1	20103	64	IGHV3-23*01	IGHD2-8*02	IGHJ6*02	ARDGGVSYYGMDV	IGLV8-61*01	IGLJ3*02	VLYMGSGIWV	NA
2	20103	64	IGHV4-38-2*02	IGHD6-6*01	IGHJ4*02	ARDPYSSSSAAFHY	IGLV8-61*01	IGLJ3*02	VLYMGSGISV	IGHM
3	20103	64	IGHV3-48*03	IGHD3-22*01	IGHJ4*02	ARAGEATYYYDSSGYYGLHNMYYFDY	IGLV8-61*01	IGLJ3*02	VLYMGSGINWV	IGHM
4	20103	64	IGHV3-30-3*01	IGHD2-15*01	IGHJ6*02	ARDQAPYCSGGSCYDASYYYYGMDV	IGLV8-61*01	IGLJ3*02	VLYMGSGISV	IGHM
5	20103	64	IGHV4-61*01	IGHD2-21*02	IGHJ4*02	ATFATALPGAYFDY	IGLV8-61*01	IGLJ2*01	VLYMNDGVVL	IGHM
6	20211	BL	IGHV3-11*05	IGHD4-17*01	IGHJ4*02	ARLDLFYGDHFDY	IGLV8-61*01	IGLJ3*02	VLYMGSGPHWV	IGHM
7	20211	64	IGHV3-21*01	NA	IGHJ3*02	ARVRDPDAFDI	IGLV8-61*01	IGLJ2*01	LLYLGSGML	IGHD
8	20211	64	IGHV4-61*02	IGHD3-3*01	IGHJ4*02	ARTTIFGVAIDY	IGLV8-61*91	IGLJ3*02	VLYMGSGINWV	IGHG1
9	20212	64	IGHV3-74*01	IGHD2-15*01	IGHJ2*01	ARDSGGWYFDL	IGLV8-61*01	IGLJ3*02	VLYMGSGIWV	IGHM
10	20217	BL	IGHV3-21*01	IGHD6-6*01	IGHJ4*02	ARGSEAARPAEIDY	IGLV8-61*01	IGLJ2*01	LLYLGSGML	IGHG1
11	20217	BL	IGHV3-30*18	IGHD3-22*01	IGHJ3*02	AKDNYYEDAFDI	IGLV8-61*01	IGLJ2*01	LLYLGSGML	NA
12	20217	64	IGHV3-49*04	NA	IGHJ1*01	TRDHWH	IGLV8-61*01	IGLJ2*01	LLYLGSGML	IGHM
13	20219	64	IGHV3-49*04	IGHD4-11*01	IGHJ6*02	TRDPESYSNPWPSADLVSHYYGMDV	IGLV8-61*01	IGLJ2*01	LLYLGSGML	IGHD
14	20219	64	IGHV3-23*01	NA	IGHJ4*02	ANRNYYFVY	IGLV8-61*01	IGLJ1*01	GLYMGSGVSV	IGHM
15	20219	64	IGHV4-30-4*09	IGHD4-11*01	IGHJ4*02	ARVRIYSDFYFDY	IGLV8-61*01	IGLJ3*02	VLYMGSDTWV	IGHM
16	20219	64	IGHV3-33*01	IGHD3-16*01	IGHJ3*02	ARDSLHSNYVYGTQFQHERIFDI	IGLV8-61*01	IGLJ3*02	VLYMGSGIWV	IGHD

Table S5. Flow cytometry panel used in this study including target, clone, fluorochrome, catalogue number and supplier.

Target	Clone	Fluorochrome	Catalogue number	Supplier
CD3	SP34-2	BV786	563918	BD
CD5	UCHT2	APC-Cy7	563516	BD
CD8	SKI	BV510	563919	BD
CD14	M5E2	RB705	757509	BD
CD19	HIBI9	BV650	302238	Biolegend
CD27	M-T271	AF700	356416	Biolegend
CD38	HIT2	BV711	563965	BD
CD43	1G10	BV421	562916	BD
CD204	U23-56	RB613	758361	BD
FCMR	HM7	APC	398104	Biolegend
IgM	G20-127	PE	555783	BD
IgD	1A6-2	RB780	569140	BD

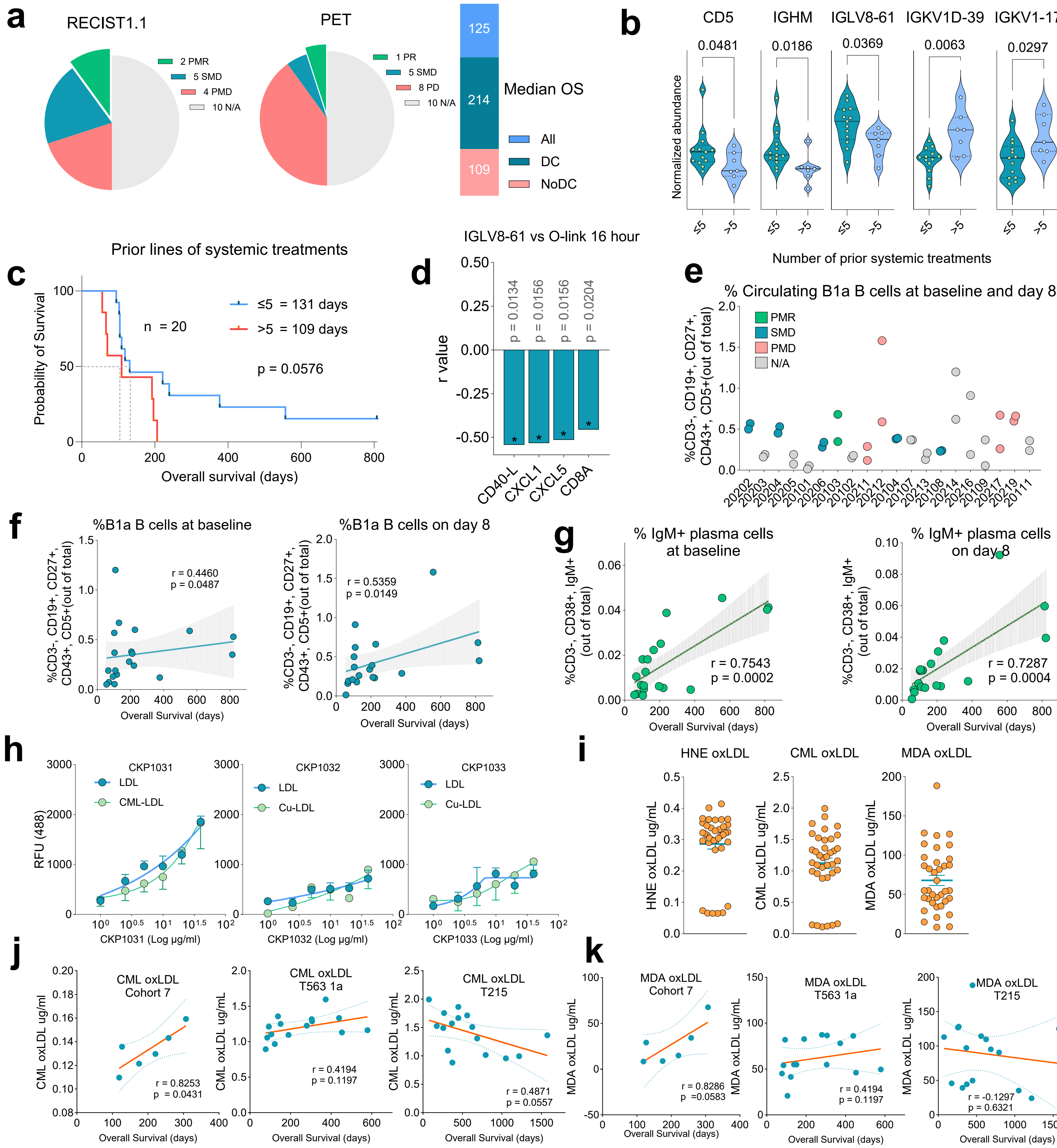

Figure S1. a. Summary of responses in the TUNIMO (T115) monotherapy trial according RECIST1.1, PET criteria and median overall survival for all patients, patients with DC and no DC. b. Group analysis identifying immunoglobulins most significantly associated with patients having more than five versus five or fewer prior lines of systemic treatment. c. Kaplan-Meier curve illustrating OS stratified by patients with more than five versus five or fewer prior lines of systemic treatment (n = 20). Statistical significance was assessed using the log-rank (Mantel-Cox) test. d. Significant correlations between IGLV8-61 abundance and NPX values of immuno-oncology-related proteins on day 8, as measured by Olink analysis. e. Flow cytometry summary showing the percentage of circulating B1a B cells ($CD3^-$, $CD19^+$, $CD27^+$, $CD43^+$, $CD5^+$) among total cells in T115 patients at baseline and day 8, with data points color-coded by best response as defined by PET criteria. f. Correlation between the percentage of circulating B1a B cells at baseline and day 8 with OS. g. Correlation between the percentage of circulating IgM+ plasma cells at baseline and day 8 with OS. h.Direct ELISA performed using IgM clones CPK1031, CPK1032, and CPK1033, with native LDL and CML modified oxLDL. i. Quantification of baseline serum concentrations of HNE oxLDL, CML oxLDL, and MDA oxLDL in patients from cohorts T115 (Cohort 7), T215, and T563 (1a). j-k. Correlation of CML oxLDL and MDA oxLDL levels with OS in patients from the TUNIMO Cohort 7, PROTA (T563 1a), and TUNITIL (T215). Cohort 7 n = 6, T563 1a n = 15, T215 n = 17.

CKP1031 Signal peptide – variable region – Constant region

Heavy Chain IgM, amino acid sequence:

MGWSCIILFLVATATGVHS
QVQLQESGPGLAKPSETLSLTCAVSGYSLSSGYYWGWIRQPPGKGLEWIGNIDHSANSYYNPSLKSRVTISVDTSKNEFSLKVRSVTA
ADTAIYYCARDPYSSSSAAFHYWGQGALVTVSS
GSASAPTLFPLVSCENSPSDTSSVAVGCLAQDFLPDSITFSWKYKNNSDISSTRGFPSVLRGGKYAATSQVLLPSKDVMQGTDEHVVC
KVQHPNGNKEKNVPLPVIAELPPKVSVFVPPRDGFFGNPRKSKLICQATGFSPRQIQVSWLREGKQVGSGVTTDQVQAEAKESGPTT
YKVTSTLTIKESDWLGQSMFTCRVDHRGLTFQQNASSMCVPDQDTAIRVFAIPPSFASIFLTKSTKLTCLVTDLTTYDSVTISWTRQNGE
AVKTHTNISESHPNATFSAVGEASICEDDWNSGERFTCTVTHTDLPSPLKQTISRPKGVALHRPDVYLLPPAREQLNLRESATITCLVTG
FSPADVFVQWMQRGQPLSPEKYVTSAPMPEPQAPGRYFAHSILTVSEEEWNTGETYTCVVAHEALPNRVTERTVDKSTGKPTLYNVS
LVMSDTAGTCY

J chain, amino acid sequence:

MGWSCIILFLVATATGVHS
QEDERIVLVDNKCKCARITSRIIRSSEDPNEDIVERNIRIIVPLNNRENISDPTSPLRTRFVYHLSDLCKKCDPTEVELDNQIVTATQSNIC
DEDSATETCYTYDRNKCYTAVVPLVYGGETKMVETALTPDACYPD

Light chain Lamda, amino acid sequence:

MGWSCIILFLVATATGVHS
QTVVTQEPSFSVSPGGTVTLTCGLSSGSVSTNYYPSWYQQTPGQAPRTLIYSTSIRSSGVPDRFSGSILGNKAALTITGAQADDEAHYY
CVLYMGSGISVFGGGTKLTVL
GQPKAAPSVTLFPPSSEELQANKATLVCLISDFYPGAVTVAWKADSSPVKAGVETTTPSKQSNNKYAASSYLSLTPEQWKSHRSYSCQ
VTHEGSTVEKTVAPTECS

CKP1032

Heavy Chain IgM, amino acid sequence:

MGWSCIILFLVATATGVHS
EVQLVESGGGLVQPGGSLRLSCAASGFTFDSYEMNWVRQAPGKGLEWVSYISSSGSTIYYADSVKGRFTISRDNAKNSLYLQMNSLR
AEDTAVYYCARAGEATYYYDSSGYYGLHNMYYFDYWGQGTLVTVSS
GSASAPTLFPLVSCENSPSDTSSVAVGCLAQDFLPDSITFSWKYKNNSDISSTRGFPSVLRGGKYAATSQVLLPSKDVMQGTDEHVVC
KVQHPNGNKEKNVPLPVIAELPPKVSVFVPPRDGFFGNPRKSKLICQATGFSPRQIQVSWLREGKQVGSGVTTDQVQAEAKESGPTTY
KVTSTLTIKESDWLGQSMFTCRVDHRGLTFQQNASSMCVPDQDTAIRVFAIPPSFASIFLTKSTKLTCLVTDLTTYDSVTISWTRQNGEA
VKTHTNISESHPNATFSAVGEASICEDDWNSGERFTCTVTHTDLPSPLKQTISRPKGVALHRPDVYLLPPAREQLNLRESATITCLVTGF
SPADVFVQWMQRGQPLSPEKYVTSAPMPEPQAPGRYFAHSILTVSEEEWNTGETYTCVVAHEALPNRVTERTVDKSTGKPTLYNVSL
VMSDTAGTCY

J chain, amino acid sequence:

MGWSCIILFLVATATGVHS
QEDERIVLVDNKCKCARITSRIIRSSEDPNEDIVERNIRIIVPLNNRENISDPTSPLRTRFVYHLSDLCKKCDPTEVELDNQIVTATQSNICD
EDSATETCYTYDRNKCYTAVVPLVYGGETKMVETALTPDACYP

Light chain Lamda, amino acid sequence:

MGWSCIILFLVATATGVHS
QTVVTQEPSFSVSPGGTVTLTCGLSSGSVSTSYYPSWYQQTPGQAPRTLIYSTNTRSSGVPDRFSGSILGNKAALTITGAQADDESDY
YCVLYMGSGINWVFGGGTKLTVL
GQPKAAPSVTLFPPSSEELQANKATLVCLISDFYPGAVTVAWKADSSPVKAGVETTTPSKQSNNKYAASSYLSLTPEQWKSHRSYSCQ
VTHEGSTVEKTVAPTECS

CKP1033

Heavy Chain IgM, amino acid sequence:

MGWSCIILFLVATATGVHS
QVQLQESGPGLVNPSETLSLSCSVSSVSVSSGNYYWSWIRQPPGKGLEWIGYMSYSGSTTYNPSLKSRVTISLDTSKNQFSLNLNSVT
AADTAVYYCATFATALPGAYFDYWGQGSLVTVSS
GSASAPTLFPLVSCENSPSDTSSVAVGCLAQDFLPDSITFSWKYKNNSDISSTRGFPSVLRGGKYAATSQVLLPSKDVMQGTDEHVVC
KVQHPNGNKEKNVPLPVIAELPPKVSVFVPPRDGFFGNPRKSKLICQATGFSPRQIQVSWLREGKQVGSGVTTDQVQAEAKESGPTTY
KVTSTLTIKESDWLGQSMFTCRVDHRGLTFQQNASSMCVPDQDTAIRVFAIPPSFASIFLTKSTKLTCLVTDLTTYDSVTISWTRQNGEA
VKTHTNISESHPNATFSAVGEASICEDDWNSGERFTCTVTHTDLPSPLKQTISRPKGVALHRPDVYLLPPAREQLNLRESATITCLVTGF
SPADVFVQWMQRGQPLSPEKYVTSAPMPEPQAPGRYFAHSILTVSEEEWNTGETYTCVVAHEALPNRVTERTVDKSTGKPTLYNVSL
VMSDTAGTCY

J chain, amino acid sequence:

MGWSCIILFLVATATGVHS
QEDERIVLVDNKCKCARITSRIIRSSEDPNEDIVERNIRIIVPLNNRENISDPTSPLRTRFVYHLSDLCKKCDPTEVELDNQIVTATQSNICDE
DSATETCYTYDRNKCYTAVVPLVYGGETKMVETALTPDACYPD

Light chain Lamda, amino acid sequence:

MGWSCIILFLVATATGVHS
QTVVTQEPSFSVSPGGTVTLTCGLRSGSVSRNSYPSWYQQMPGQAPRTLIYNTNTRSSGVPHRFSGSILGNKAALTITGAQADDESDY
YCVLYMNDGVVLFGGGTKLTVL
GQPKAAPSVTLFPPSSEELQANKATLVCLISDFYPGAVTVAWKADSSPVKAGVETTTPSKQSNNKYAASSYLSLTPEQWKSHRSYSCQ
VTHEGSTVEKTVAPTECS

Figure S2. Full length sequences used to synthesize the pentameric IgMs CKP1031, CKP1032 and CKP1033. The amino acid sequence for the heavy chain, J chain and light chain are coloured according to the signal peptide (blue), variable region (green) and constant region (black).

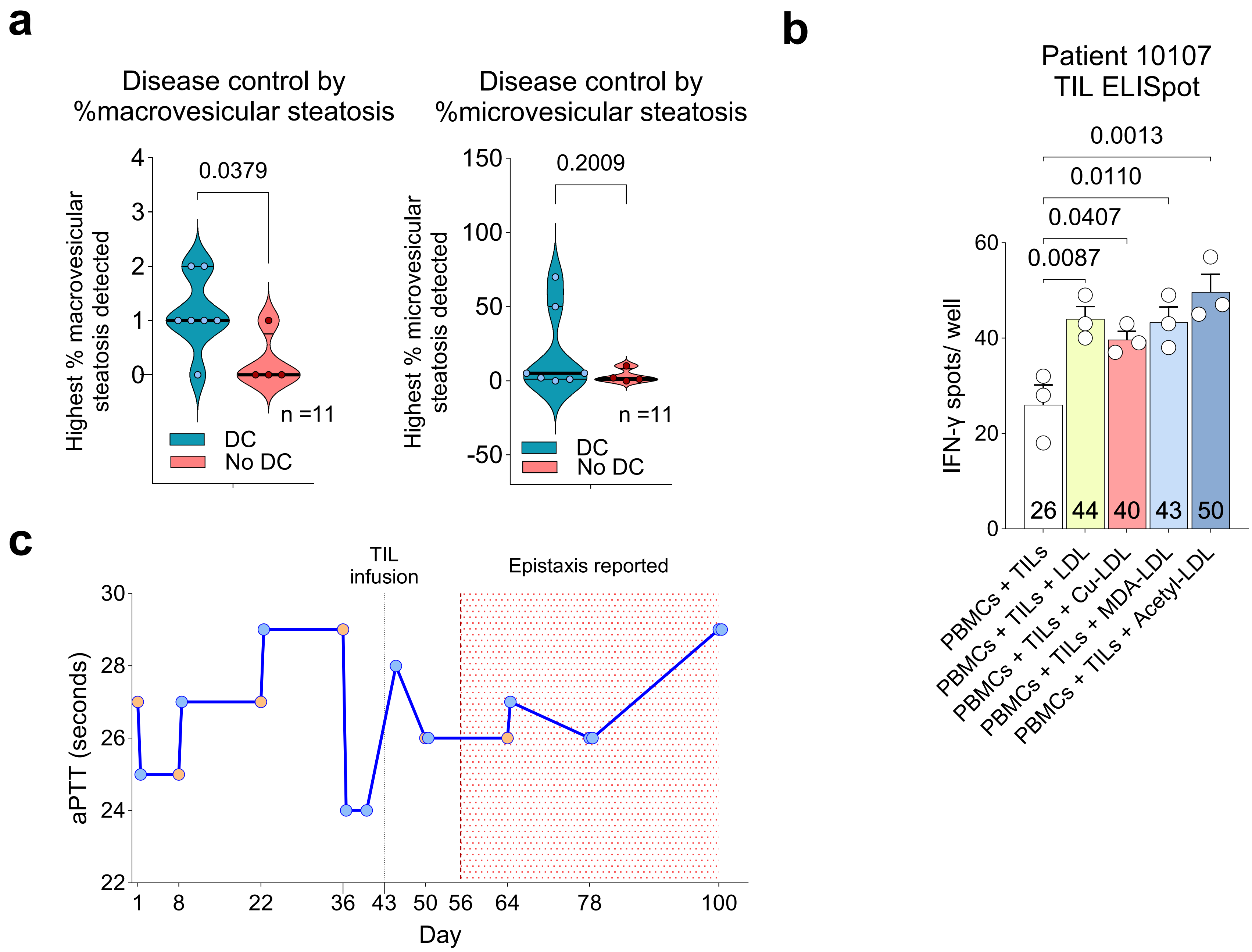

Figure S3. a. Comparison of pathologist-assessed percentages of macrovesicular and microvesicular steatosis in liver biopsies from patients with and without DC. b. Human IFN-γ ELISpot assay assessing the reactivity of the TIL infusion product cultured with PBMCs (baseline) from patient 10107, enrolled in the T215 trial, against native and modified forms (Cu-LDL, MDA-LDL and Acetyl-LDL) of LDL. Mean number of spots is indicated at the bottom of each bar. c. aPTT duration recorded during treatment of patient 10107, with the day of TIL infusion indicated by a black dotted line and the onset of epistaxis marked by a red dotted line. Statistical significance in a. was determined using a two-tailed Mann-Whitney U test. For panel b., significance was assessed using Dunnett's test, with error bars representing the standard error of the mean (SEM).
